# Normative trajectories of R _1_ , R _2_ *, and magnetic susceptibility in basal ganglia on healthy ageing

**DOI:** 10.1162/imag_a_00456

**Published:** 2025-02-03

**Authors:** Kwok-Shing Chan, Marcel P. Zwiers, Michelle G. Jansen, Martin E. Johansson, Rick C. Helmich, Joukje M. Oosterman, David G. Norris, Christian F. Beckmann, José P. Marques

**Affiliations:** Donders Institute for Brain, Cognition and Behaviour, Radboud University, Nijmegen, The Netherlands; Department of Cognitive Neuroscience, Radboud University Medical Center, Nijmegen, The Netherlands; Athinoula A. Martinos Center for Biomedical Imaging, Massachusetts General Hospital, Charlestown, MA, United States; Department of Radiology, Harvard Medical School, Boston, MA, United States; Neurology Department, Donders Institute for Brain, Cognition and Behaviour, Radboud University Medical Centre, Nijmegen, The Netherlands; The Erwin L. Hahn Institute, University of Duisburg-Essen, Essen, Germany; MIRA Institute for Biomedical Technology and Technical Medicine, University of Twente, Twente, The Netherlands

**Keywords:** quantitative MRI, normative modelling, ageing trajectories, magnetic susceptibility, relaxometry, iron, myelin, Parkinson disease

## Abstract

Quantitative MRI (qMRI) techniques, including R_1_, R_2_*, and magnetic susceptibility mapping, have emerged as promising tools for generating surrogate imaging markers of brain tissue microstructure, enabling non-invasive in vivo measurements associated with myelination and iron deposition. Gaining insights into how these quantitative measurements evolve throughout a normal lifespan can enhance our understanding of brain maturation processes and facilitate studies of disease-related microstructural changes by distinguishing pathological alterations from normal brain development. In this study, we established the normative trajectories of R_1_, R_2_*, and magnetic susceptibility in the basal ganglia at 3T. We used a healthy ageing cohort comprising 260 subjects with an evenly distributed age range (from 18 to 80 years) and sex ratio throughout adulthood. Utilising the non-parametric Gaussian Process Regression model to derive the normative trajectories, we found that R_1_in these structures predominantly exhibits a quadratic shape over age, while R_2_* and magnetic susceptibility are primarily linear. We validated the normative trajectories of R_2_* and magnetic susceptibility using an independent cohort. This result reinforces existing findings on the association between age and qMRI. Additionally, we demonstrated that the spatial distributions of the qMRI parameters also change with age in the putamen and caudate nucleus. Finally, the utility of normative modelling of qMRI in the basal ganglia is validated using an independent cohort comprising both healthy participants and individuals with Parkinson’s disease, with comparable data acquisition protocols.

## Abbreviations

Cau: Caudate nucleus
Pu: Putamen
GPe: External globus pallidus
GPi: Internal globus pallidus
NAcc: Nucleus accumbens
SNr: Substantia nigra pars reticulata
SNc: Substantia nigra pars compacta
RN: Red nucleus
VP: Ventral pallidum
STN: Subthalamic nucleus

## 1 Introduction

Studying brain development across the lifespan is crucial for understanding brain maturation and establishing a standard framework to help us evaluate neurological disorder-induced deviations from typical quantitative phenotypes at an individual level. Traditional studies often involve group comparisons, such as between healthy individuals and patients, to distinguish pathological features from normal brain development, necessitating matching group demographic characteristics. Factors such as age and sex are known to influence various brain (micro)structures (Gur et al., 2002;Jäncke et al., 2015;Persson et al., 2015;Xu et al., 2000). However, simply matching demographic characteristics may not be sufficient, as this overlooks how these factors might influence the effect of interest. The development of normative models allows for the exploration of deviations related to diseases from what is considered normal (Marquand, Rezek, et al., 2016;Rutherford et al., 2023), considering their dependence on these factors. This approach transcends classical case–control designs, allowing researchers to better comprehend disease progression and the effects of medical interventions without the need for acquiring prohibitively large control datasets due to cost and logistical constraints.

Most of the existing literature on normative MRI data focuses primarily on changes in brain structure, such as variations in tissue volume and cortical thickness (Bethlehem et al., 2022;Ramanoël et al., 2018;Scahill et al., 2003;Terribilli et al., 2011). This emphasis on morphometric characteristics stems from the relative nature of the underlying MR signal rendering the intensity of imaging voxels arbitrary, and the growing availability of conventional structural images across various large public datasets (Bookheimer et al., 2019;Jack et al., 2008;Miller et al., 2016;Satterthwaite et al., 2016;Snoek et al., 2021). Prior investigations have indicated a rapid increase in cortical thickness and tissue volumes during the early stages of development, followed by a gradual decrease after 1–2 years for cortical thickness and 18 years for total tissue volume (Bethlehem et al., 2022). Furthermore, studies have observed morphological disparities related to sex and hemispheric asymmetry (Bethlehem et al., 2022;Park et al., 2004;Taki et al., 2011;Watkins et al., 2001). In addition to cortical thickness, the framework of normative modelling can be expanded to encompass various other imaging-derived phenotypes, including diffusion-derived metrics in white matter among the most prevalent and quantitative MRI (qMRI) properties.

Quantitative MRI parameters, including the longitudinal relaxation rate R_1_, apparent transverse relaxation rate R_2_*, and tissue magnetic susceptibility*χ*, are gaining prominence as surrogate biomarkers for myelin (Lutti et al., 2014;Rooney et al., 2007) and iron (Ghadery et al., 2015;Langkammer et al., 2012;Pontillo et al., 2022;Stüber et al., 2014) concentrations. Quantitative MRI offers direct and quantitative assessments of tissue MR properties. This approach not only holds the promise of furnishing more precise information about brain tissue composition but also of generating metrics that are reproducible across sessions and scanners (Deh et al., 2015;Körzdörfer et al., 2019;Leutritz et al., 2020;Lin et al., 2015;Teixeira et al., 2020). These methods are becoming more readily available in MRI scanners and cohort datasets. Their reproducibility and sensitivity have prompted various studies demonstrating that R_1_, R_2_*, and*χ*vary throughout the lifespan (Kühne et al., 2021;G. Li et al., 2023;W. Li et al., 2014;Miletić et al., 2022), with these changes being specific to regions of interest (ROI). In traditional age-matched case–control studies, these metrics are significantly affected in various brain regions depending on the diseases, reflecting, for example, increased iron deposition in the putamen and substantia nigra in Alzheimer’s and Parkinson’s diseases (Acosta-Cabronero et al., 2013;Pyatigorskaya et al., 2020), respectively.

Summary statistics, such as mean or median, are widely used to study changes in qMRI through ROI-based analysis (Seiler et al., 2020). Previous research has outlined normative trajectories in adulthood, showing an inverted U-shape for R_1_(Erramuzpe et al., 2020;Yeatman et al., 2014) and linearity for R_2_* and*χ*(Acosta-Cabronero et al., 2016;Burgetova et al., 2021;Keuken et al., 2017;Y. Li et al., 2021). Notably, recent findings suggest that both ageing and diseases can impact the spatial distribution of these quantitative parameters (Drori et al., 2022;Oldehinkel et al., 2022) and manifest in higher order statistical analysis (G. Li et al., 2019). Alternatively, normative modelling of qMRI can be conducted at the voxel level, as exemplified in the work ofPiredda et al. (2020,^2023^). These quantitative atlases serve as valuable resources for facilitating automated pathology detection at the voxel level. However, this approach mandates high-quality non-linear image registration to align individual data with the atlases, a task compounded by individual-level morphological differences, especially in cortical grey matter studies, where structural variability is increased. For instance, the primary auditory cortex can consist of 1 or 2 Heschl’s gyri (Costa et al., 2011). Moreover, the creation of the normative image template is more prone to measurement noise due to the lower signal-to-noise ratio at the voxel level and imaging artefacts in contrast to ROI-based analysis. All these sources of variance will reduce the sensitivity of using the normative template in detecting subtle changes.

In this work, we aimed to explore both the cross-sectional median and spatial effects of age on qMRI parameters R_1_, R_2_*, and*χ*in the basal ganglia at 3T, utilising a healthy ageing cohort characterised by a broad age distribution. Our focus on subcortical nuclei stems from their robustness in*χ*measurements and the well-established association with iron accumulation. Our objectives were threefold: (1) to delineate normative trajectories across subcortical grey matter nuclei throughout the lifespan using a Gaussian Process Regression model along with conventional volumetric analysis; (2) to investigate the independence of deviations from normative values within each subcortical nucleus, as measured by different qMRI parameters; and (3) to examine the independence of variations from normative values across the subcortical nuclei, aiming to elucidate networks of co-variation from the norm. Lastly, we tested the utility of the normative trajectories in a validation dataset consisting of both healthy participants and people with Parkinson’s disease, a disorder that has been associated with increased iron deposition in the substantia nigra (Biondetti et al., 2021;Mitchell et al., 2021).

## 2 Material and Methods

### 2.1 Data acquisition

Data utilised to derive the normative trajectories is part of the Advanced Brain Imaging on Ageing and Memory (ABRIM) data collection (Jansen et al., 2024) available athttps://doi.org/10.34973/7q0a-vj19. A complete description of the MRI protocol and the pre-processing steps can be found inJansen et al. (2024). Briefly, data acquisition was performed at 3T (Siemens, Erlangen, Germany) on 301 healthy volunteers. Visual data curation was performed using an in-house developed tool*“slicereport”*of BIDScoin (Zwiers et al., 2022). Three raters (M.J., K.C., and M.R.) visually assessed the quality of all datasets. The following criteria were used to exclude data from further analysis:

- Does the imaging volume cover the whole brain, including the cerebellum? (see, e.g., Subject 24 inSupplementary Fig. S2)
- Does brain extraction apply correctly?
- Any visible motion artefacts, such as strong ringing artefacts and low-frequency fluctuations on white matter that are not morphologically feasible and can severely affect the quantitative maps (particularly R_2_* maps)? (see Subject 25 inSupplementary Fig. S2)

Forty-one participants were excluded due to incidental findings and/or did not fulfil the aforementioned criteria, resulting in data of 260 participants to be included in the statistical analysis (18–79 years; mean ± standard deviation (SD) = 50.8 ± 16.8 years, seeTable 1andSupplementary Fig. S1). Informed consents were obtained from all participants included in this study in accordance with the Declaration of Helsinki. The imaging protocol of the data analysed here consisted of

1. Whole-brain T_1_(= 1/R_1_) scan using MP2RAGE (Marques et al., 2010), α_1_/α_2_= 6°/6°, TI_1_/TI_2_= 700 ms/2400 ms, TR/TE = 6000 ms/2.34 ms, resolution = 1 mm iso., acquisition time (TA) = 7 min;
2. Whole-brain turbo FLASH B_1_mapping, TR/TE = 10000/2.23, α_SAT_/α = 80°/8°, resolution = 3.3 mm x 3.3 mm x 2.5 mm, TA = 20 s;
3. Bipolar 3D multi-echo GRE, TR/TE1/ΔTE = 44 ms/6.14 ms/4 ms, 9 echoes, GRAPPA = 3, phase/slice partial Fourier = 0.875/0.875, α = 20°, resolution = 0.8 mm iso., TA = 9.5 min.

**Table 1.** Demographics of the ABRIM dataset used in this work.

		Age range (years)					
ABRIM	Full range	18–30	31–40	41–50	51–60	61–70	71–79
N	260	39	41	44	49	43	44
Age mean ± SD (years)	50.8 ± 16.8	24.6 ± 3.9	35.4 ± 3.0	46.0 ± 2.9	55.4 ± 2.9	65.2 ± 3.0	74.0 ± 2.5
N _Male_	119	17	17	21	24	22	18
Age mean ± SD (years)	51.2 ± 16.2	26.3 ± 3.1	34.3 ± 3.1	46.0 ± 3.2	55.4 ± 2.8	65.4 ± 3.0	73.7 ± 2.6
N _Female_	141	22	24	23	25	21	26
Age mean ± SD (years)	50.5 ± 17.4	23.4 ± 4.1	36.1 ± 2.8	46.0 ± 2.7	55.5 ± 3.0	65 ± 3.0	74.1 ± 2.6

To study the applicability of the R_2_* and*χ*normative models derived from the ABRIM datasets, an independent cohort with a similar imaging protocol, which is part of the “Personalized Parkinson Project” (PPP) data collection (Bloem et al., 2019), was used. Briefly, the PPP study followed 520 patients who were diagnosed with Parkinson’s disease for 2 years, aiming to gain insight into the course of Parkinson’s disease, as well as 60 healthy age- and sex-matched controls. All patients were within 5 years of diagnosis and full clinical characteristics can be found inJohansson, Toni, et al. (2023)andJohansson, van Lier, et al. (2023). During the study, two MRI scans were made for each participant, both at baseline and after 2 years. The same visual data curation process used for the ABRIM dataset was applied to assess the quality of the PPP dataset. For the patient data, only the first scan was included in the subsequent analysis. Similarly, for the healthy volunteer data, only one scan from the two MRI visits was included. If the first scan did not meet the inclusion criteria, the second scan was included, provided it fulfilled the visual inspection criteria and we assumed there is no disease-related progression between the two scans for the healthy control data. In this analysis, data from 44 healthy volunteers and 316 patients were used. The demographics are shown inTable 2andSupplementary Figure S1. Data acquisition was performed at 3T (Siemens, Erlangen, Germany). The imaging protocol of the data analysed here consisted of

**Table 2.** Demographics of the PPP datasets used in this work.

PPP	Control	Patient
N (Male; Female)	44 (20; 24)	316 (175; 141)
Age mean ± SD (years)	58.2 ± 9.2 (60.3 ± 8.7; 56.4 ± 9.5)	60.9 ± 9.0 (60.8 ± 9.1; 60.9 ± 8.9)

1. Whole-brain T_1_-weighted anatomical scan using MPRAGE, α = 8°, TI = 880 ms, TR/TE = 2000 ms/2.03 ms, resolution = 1 mm iso., TA = 5 min;
2. Bipolar 3D multi-echo GRE, TR/TE1/ΔTE = 44 ms/6.14 ms/4 ms, 9 echoes, GRAPPA = 3, phase/slice partial Fourier = 0.875/0.75, α = 20°, resolution = 0.8 mm iso., TA = 8 min.
  where TI, TR, TE and TA stand for inversion, repetition, echo, and acquisition times, respectively.

### 2.2 Data analysis

#### 2.2.1 Data processing

SynthStrip (Hoopes et al., 2022) was used for skull stripping on the second inversion time of the R_1_data (INV2, proton density contrast) with the ABRIM cohort or the MPRAGE image with the PPP cohort, and the first echo of the GRE data (GRE1). For*χ*processing, the GRE brain masks were first refined by excluding voxels with high R_2_* values on the brain mask edge to improve estimation robustness.*χ*maps were derived using SEPIA v1.2.2.4 (Chan & Marques, 2021) with the following pipeline: bipolar gradient phase correction (J. Li et al., 2015), ROMEO (Dymerska et al., 2021) for total field computation, V-SHARP (W. Li et al., 2011) for background field removal, and LP-CNN (Lai et al., 2020) for dipole field inversion. A retrospective correction method was implemented to correct those datasets acquired with a tilted field-of-view with respect to the main magnetic field direction before the dipole field inversion step (Kiersnowski et al., 2023). The mean susceptibility value across the whole brain was used as a reference. For R_2_* mapping, Marchenko–Pastur Principal Component Analysis (MP-PCA) denoising (Veraart et al., 2016) with a patch size of 5 x 5 x 5 voxels was applied to the complex-valued mGRE data to improve SNR and the magnitude of the denoised data was then extracted to derive R_2_* maps based on a closed-form solution (Gil et al., 2016).

Quantitative R_1_maps were obtained using the BIDS-compatible Matlab function*“bids_T1B1correct.m*” hosted on GitHub (https://github.com/Donders-Institute/MP2RAGE-related-scripts). This function performs (1) registration of the magnitude image from the turbo FLASH B_1_map to the MP2RAGE image with INV2 using SPM12 (Penny et al., 2006); (2) the resulting transform matrix was used to register a spatially smoothed version of the B_1_^+^map to the MP2RAGE space, which was then used to (3) correct for the B_1_transmit inhomogeneities in R_1_estimations (and derive M_0_maps over a wide range of R_1_values). In the last step, a fingerprinting-like approach (Ma et al., 2013) was used to estimate R_1_instead of the traditional MP2RAGE lookup table (Marques et al., 2010). Readers may refer to the Supplemental Material andFigure S5for a detailed example of the differences between the use of dictionary matching and the conventional MP2RAGE lookup table. Dictionary matching was achieved by searching the maximum likelihood of the inner product of the signal at the two inversion times by a dictionary of simulated signals across a predefined range of R_1_and M_0_values as implemented in the function*“MP2RAGE_dictionaryMatching.m.”*The MP2RAGE signal dictionaries were generated with a step size of 0.005 nominal B_1_field in order to account for transmit field inhomogeneities.

#### 2.2.2 Subcortical grey matter parcellation

All data and statistical analyses were conducted in the common MNI space (ICBM 2009c Nonlinear Asymmetric) (Fonov et al., 2009). The entire image registration procedures are shown inFigure 1a. The MuSus-100 atlas (He et al., 2022) was used to provide parcellation labels of the basal ganglia, including detailed thalamic sub-regions. Co-registration between R_1_and GRE data was achieved using a rigid body transformation between the skull-stripped MP2RAGE’s INV2 image and the GRE1 image. This transformation matrix was used to transform the*χ*and R_2_* maps into the R_1_space for each subject. To facilitate high-quality subcortical grey matter parcellation, a four-step registration procedure was performed as follows.

**Fig. 1.**
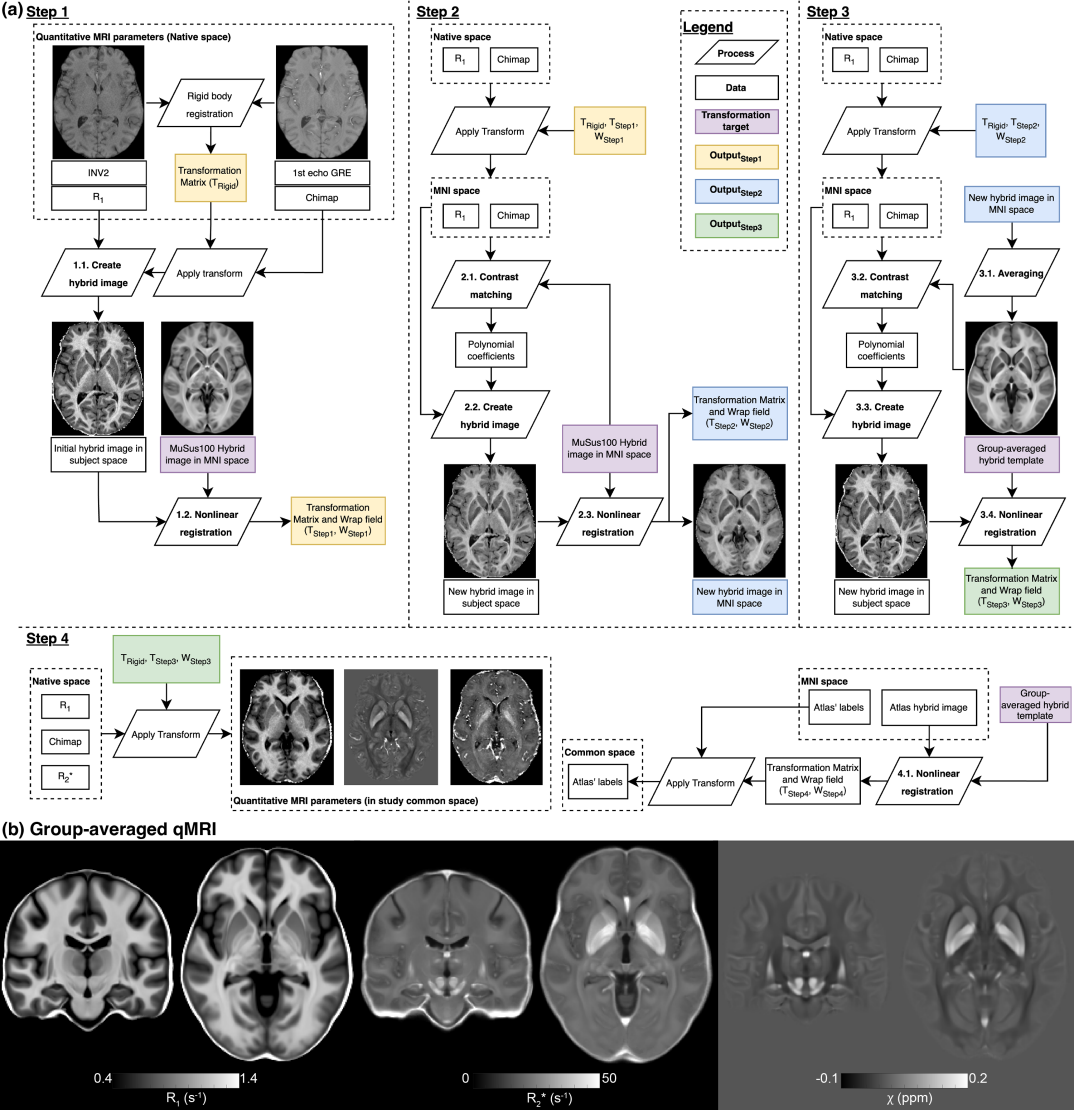
(a) An illustration of the image registration process on the three qMRI parameters maps. (b) Example R_1_, R_2_*, and*χ*maps derived by averaging data across all subjects in the study common space.

*Step 1:*The first step involved the creation of an initial R_1_-*χ*hybrid image, which was then registered to the MuSus-100 atlas based on nonlinear transformation (Avants et al., 2011). The hybrid image was created using the following equation (Zhang et al., 2018):

$$hybrid=\mu_{0}+\mu_{1}\chi +\mu_{2}R_{1,norm},$$ (1)

where R_1,norm_is the R_1_map normalised to values between 0 and 255 (clipping 1% brain-masked values on each side of the histogram).$\mu_{0}$,$\mu_{1},$and$\mu_{2}$were set to 0, 400, and 1 according toZhang et al. (2018). Since the hybrid image was derived from the R_1_map here instead of the T_1_-weighted MPRAGE image and a different algorithm was used to derive the*χ*map, the contrast of the initial hybrid image does not match exactly the hybrid template of the atlas (e.g., see the similar contrast between external globus pallidus and putamen inSupplementary Fig. S3), but the improved contrasts between brain tissues do provide a fair alignment between the subject’s data and the atlas data. This procedure has now been integrated into the SEPIA toolbox as of version 1.2.2.4.

*Step 2:*The second step involved creating a hybrid image closely resembling the atlas template. To achieve this, we performed image contrast matching to enhance the similarity in image intensity between the subject’s R_1_map and the atlas’s T_1_w template. This was performed by fitting a second-order polynomial function to the median value of all ROIs in the brain based on the parcellation labels of the atlas. A similar procedure was performed on the*χ*map, but in this case, a first-order polynomial function was used. Subsequently, the polynomial coefficients inEquation (1)were derived from the atlas’s hybrid template with the matched subject’s R_1_and*χ*data, which were then used to compute a new hybrid image for nonlinear registration. This step is crucial as it significantly improves the delineation of subcortical grey matter structures, as demonstrated inSupplementary Figure S3.

*Step 3:*In the third step, a group hybrid template was created by averaging the hybrid images from all subjects derived from Step 2. We then repeated the contrast matching procedures in the second step with the new group-averaged hybrid image instead of the atlas’s template. While the change of hybrid image contrasts between the basal ganglia structures is less noticeable as in Step 2, the update of the registration target from the atlas’ template to the new group-averaged image further improves the registration quality as the new group-averaged image is derived from the same tissue contrast in our cohort data. This step further improves the registration quality between the subject data and the template, which can be seen inSupplementary Figures S3 and S4where the coefficient of variation on the final hybrid image across all subjects was reduced around the caudate nucleus/ventricle boundary when compared to the Step 2 result. All the qMRI maps were transformed into the group template space using the registration results of this step. The first three steps ensure high-quality image registration for all data to be transformed into a common space.

*Step 4:*As a final step, subcortical grey matter structure parcellation was performed by using a nonlinear image transformation to register the atlas hybrid template to the group hybrid template (Fig. 1a). For the PPP cohort, the same registration procedures were repeated within the cohort datasets to obtain the subcortical grey matter parcellation, with the only change being using the T_1_-weighted MPRAGE image instead of the R_1_map for image registration. Examples of parcellation quality can be found inSupplementary Figure S4.

#### 2.2.3 Metrics of interest

To further minimise the variations caused by imperfect image registration and potential outliers from veins in computing the normative models, the median qMRI (R_1_, R_2_*, and*χ*) values were calculated for each subcortical grey matter structure. In addition to analysing each thalamic nucleus individually, we also processed the thalamus as a whole structure by combining all thalamus-associated labels from the MuSus-100 atlas. Besides the median statistics, the interquartile range (IQR) and skewness of the distributions were computed on each subject.

The tissue volumes of all subcortical ROIs were also extracted and normalised by the intracranial brain volume. To facilitate visualisation of the evolution of different volumes on the same plots, the volume of a given ROI was normalised by the population mean ($\bar{V_{ROI}}$), and the percentage change in volume ($V_{ROI,\%}$) is studied as

$$V_{ROI,\%}=\frac{\left(V_{ROI}-\bar{V_{ROI}}\right)}{\bar{V_{ROI}}}\times 100,$$ (2)

where$V_{ROI}$is the volume normalised by the intracranial brain volume.

To study the association between age and qMRI spatial distribution on the putamen and caudate nucleus, a first-order 3D polynomial function was employed to fit the spatial distribution across the ROI mask, which was eroded by one voxel in all directions to reduce the partial volume effect at the edges of the structures, so that the spatial gradients in the lateral-medial (L-M), posterior-anterior (P-A), and inferior-superior (I-S) directions of the MNI coordinate space can also be investigated in these structures, which can be expressed as follows:

$$qMRI\left(x,y,z\right)=p_{1}\left(x-x_{0}\right)+p_{2}\left(y-y_{0}\right)+p_{3}\left(z-z_{0}\right)+p_{0},$$ (3)

where$p_{1}$,$p_{2},$and$p_{3}$describe the spatial gradient in the L-M, P-A, and I-S directions, respectively,$p_{0}$is the constant term representing the mean qMRI, and$x_{0}$,$y_{0},$and$z_{0}$are the Cartesian coordinates of the centre of mass of the ROI mask. This analysis was limited to the putamen and caudate nucleus as the size of other structures is too small with respect to the spatial resolution to have an accurate estimation of the spatial gradient, and because spatial gradients have been reported for these regions in the past (Drori et al., 2022).

#### 2.2.4 Normative modelling

##### 2.2.4.1 General linear model

General Linear Model (GLM) analysis was conducted to investigate the impact of ageing and other demographic factors on qMRI parameters in various structures (ROI). The design matrix comprised four regressors: Age, Age^2^, Sex, and (left-/right-) Hemisphere:

$$\begin{array}{l} qMRI_{median/\Delta \left(L-M\right)/\Delta \left(A-P\right)/\Delta \left(V-D\right)}=\beta_{mean}+\beta_{Age}Age\\ \;\;\;+\beta_{Age^{2}}Age^{2}+\beta_{Sex}Sex+\beta_{Hemisphere}Hemisphere. \end{array}$$ (4)

The utility of Age^2^as a regressor is to consider the inverted U-shaped effect observed in our R_1_data which were also shown previously (Yeatman et al., 2014). Z-statistics for each regressor were computed by converting the t-statistic of GLM using*fsl_glm*(Jenkinson et al., 2012), reflecting the effect of the regressor on the measured qMRI metric.

##### 2.2.4.2 Gaussian process regression

In the following normative modelling analysis, the data were corrected for the sex-specific and inter-hemispherical effects based on the GLM results. Gaussian Process Regression (GRP) (Marquand, Wolfers, et al., 2016), a non-parametric supervised learning method, from PCNToolkit (Rutherford, Kia, et al., 2022) was used to compute the normative trajectory of the sex- and hemispheric-corrected qMRI metrics as a function of age with 10-fold cross-validation. While GRP also provides the z-statistics as a major output, in contrast to GLM, the z-score reflects the degree of deviation of the input data from the normative data.

#### 2.2.5 Correlation analysis

While both iron deposition and myelin concentration in the brain can contribute to all the studied qMRI metrics, the qMRI metrics are known to have different sensitivities to the changes in the iron and myelin concentrations. To investigate whether the various qMRI metrics possess similar/redundant information, Pearson’s correlation analysis was performed on the qMRI metric z-scores derived from the GRP of all subjects for each subcortical grey matter structure. In this way, we can investigate whether the deviations from the norm in one qMRI metric are predictive of the deviations in another metric. Similarly, the associations among the subcortical grey matter structures on each qMRI metric were studied by performing Pearson’s correlation analysis on the GRP’s z-scores between the structures. With this analysis, we aimed to evaluate whether deviations from the norm are independent across ROIs, or whether there are any networks of nuclei where the relaxation values tend to jointly fluctuate.

#### 2.2.6 Validation using the PPP dataset

Validation of the normative models derived from the ABRIM data was performed by applying the normative models to the PPP cohort. Both R_2_* and*χ*in the PPP dataset were first corrected for the sex and hemispheric differences based on the GLM results of the ABRIM cohort, from which the z-statistics were computed using the GPR normative models. Two-sample t-tests were also performed to compare the z-scores and the median qMRI values of each structure between the healthy controls and Parkinson’s disease patients as a confirmatory analysis of previous findings where significantly higher values were found in substantia nigra (both R_2_* and*χ*) and in globus pallidus (only*χ*) (Langkammer et al., 2016;Pyatigorskaya et al., 2020). The Benjamini and Hochberg procedure was used to control the false discovery rate (FDR) of 0.05 (Benjamini & Hochberg, 1995;Benjamini & Yekutieli, 2001). An adjusted*p*-value of 0.05 after the FDR correction is considered statistically significant. Furthermore, we computed the normative trajectories using only the PPP healthy individual data following the same processing steps to evaluate whether the normative trajectories were comparable when data with a narrower age range were used.

## 3 Results

### 3.1 GLM analysis on age, sex, and hemispheric effects on qMRI

**Fig. 2.**
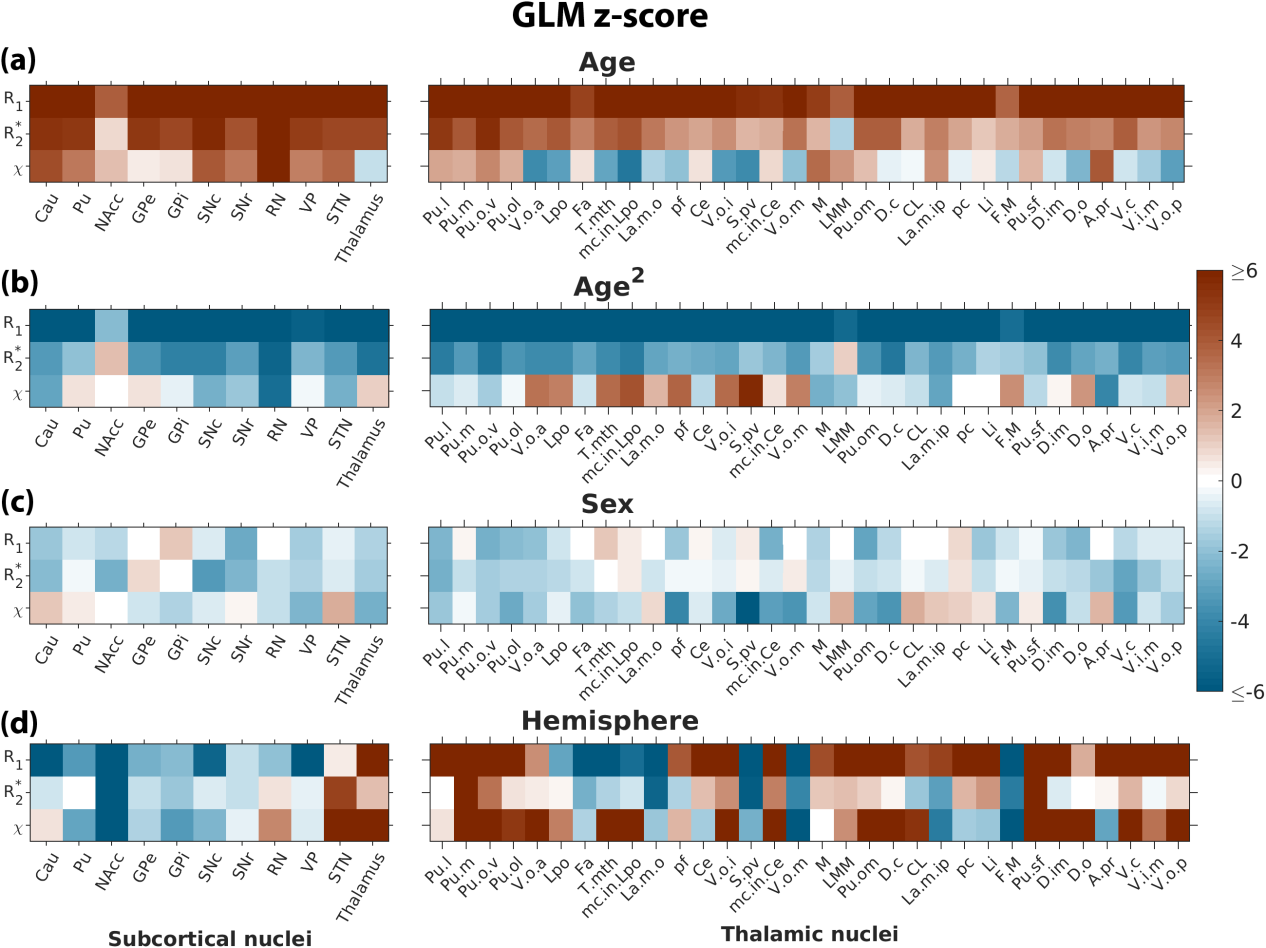
Z-scores of GLM expressed inEquation (3)for (a) Age, (b) Age^2^, (c) Sex, and (d) Hemisphere. Within each panel, the z-scores for the subcortical nuclei (left) and thalamic sub-nuclei (right) are shown. The evaluated thalamic sub-nuclei were: Pu.l - Pulvinar (Pu) thalami (th) laterale; Pu.m - Pu. th. mediale; Pu.o.v - Pul. th. orale-ventralis; Pu.ol - Pu. th. oral laterale; V.o.a - Ventrooralis anterior; Lpo - Lateropolaris th.; Fa - Fasciculosus th.; T.mth - Tractusmammillo-thalamicus; mc in LPO; La.m.o - Lamella medialis oralis; pf - Parafascicularis th.; Ce - Centralis th.; V.o.i - Ventrooralis internus; S.pv - Substantia periventricularis; mc in Ce; V.o.m - Ventrooralismedialis; M - Dorsomedialis; LMM - Lamina medullaris medialis; Pu.om - Pu. th. orale mediale; D.c - Dorso-caudalis; CL - Centralis lateralis; La.m.ip - Lamella medialis interpolaris; pc - Paracentral; Li - Limitans th.; F.M - Fasciculus retroflexus Meynertii; Pu.sf - Pu. superficiales; D.im - Dorso-intermedii; D.o - Dorso-orales thalami; A.pr - Anteroprincipalis th.; V.c - Ventrocaudalis; V.i.m - Ventrointermedius; V.o.p - Ventrooralis posterior.

Figure 2presents the z-statistics results of the GLM analysis conducted on the qMRI metrics for each subcortical ROI. Ageing (as represented by either Age or Age^2^regressor) exhibited the most statistically significant effects on the measured qMRI metrics within the subcortical and thalamic nuclei (Fig. 2a, b). Across all structures, a z-score of at least 3.86 was observed in at least one of the qMRI values on ageing. Notably, among the subcortical nuclei, the nucleus accumbens (NAcc) displayed the weakest age dependence on R_1_and R_2_*, while the internal and external globus pallidus (GPi) and (GPe), despite being highly paramagnetic, exhibited the weakest age dependence on*χ*. Conversely, red nucleus (RN) showed a strong age dependence across all three metrics. Generally, ageing manifested a positive linear effect (depicted in red,Fig. 2a) and a negative quadratic effect (depicted in blue,Fig. 2b) on the qMRI metrics for the subcortical nuclei. The Age^2^effect was more pronounced on R_1_and to a lesser extent on R_2_* across all ROIs (${\bar{\left|z_{Age^{2}}\right|}}_{R_{1}}=8.22s$vs.${\bar{\left|z_{Age^{2}}\right|}}_{R_{2}^{*}}=3.11$). Overall, among the three qMRI metrics, ageing exerted a stronger impact on R_1_than on R_2_* and*χ*($\bar{\max\left({\left|z_{Age}\right|}_{R_{1}},{\left|z_{Age2}\right|}_{R_{1}}\right)=8.37}$vs.$\bar{\max\left({\left|z_{Age}\right|}_{R_{2}^{*}},{\left|z_{Age2}\right|}_{R_{2}^{*}}\right)}$=3.57vs.$\bar{\max\left({\left|z_{Age}\right|}_{\chi },{\left|z_{Age2}\right|}_{\chi }\right)}=2.44$). Sex demonstrated the weakest effect on qMRI among the four demographic factors (Fig. 2c), while interhemispheric differences exhibited notable effects on various structures (Fig. 2d). Interhemispheric effects were particularly prominent in the NAcc and thalamic nuclei (also thalamus as a whole), with interhemispheric differences exclusive to R_1_observed in the caudate nucleus (Cau), substantia nigra pars compacta (SNc), and ventral pallidum (VP). When focusing on thalamic nuclei, similar age-dependence trends were observable for R_1_and R_2_* (positive and negative z-scores for Age and Age^2^, respectively), whereas for*χ*, the linear age dependence displayed varying polarities across nuclei.

### 3.2 GPR normative trajectories of basal ganglia

The normative trajectories on how subcortical nucleus volume, R_1_, R_2_*, and*χ*change as a function of age are shown inFigure 3. Normalised tissue volumes generally decreased with increasing age, except for VP. The majority of R_1_trajectories exhibited an inverted U-shape, whereas R_2_* and*χ*trajectories tended to be more linear, consistent with the GLM findings inFigure 2despite the non-parametric nature of the GPR analysis. Notably, NAcc and VP stood out as clear outliers from the inverted U-shaped trajectory of R_1_, displaying a parabolic behaviour that did not reach a maximum within the age range of our study. Conversely, most other nuclei appeared to reach a maximum R_1_between the ages of 45 and 60 years. Despite the flexibility of GPR, the derived trajectories supported the utilisation of the quadratic age model within the age range here (18–79 years). Most structures exhibited both different medians and rates of development across the lifespan. Overall, spatially proximate sub-structures, such as GPe and GPi, and SNc and SNr, demonstrated similar behaviour both in volume and in R_1_, while the substantia nigra sub-structures were clearly distinct in both*χ*and R_2_* trajectories.

**Fig. 3.**
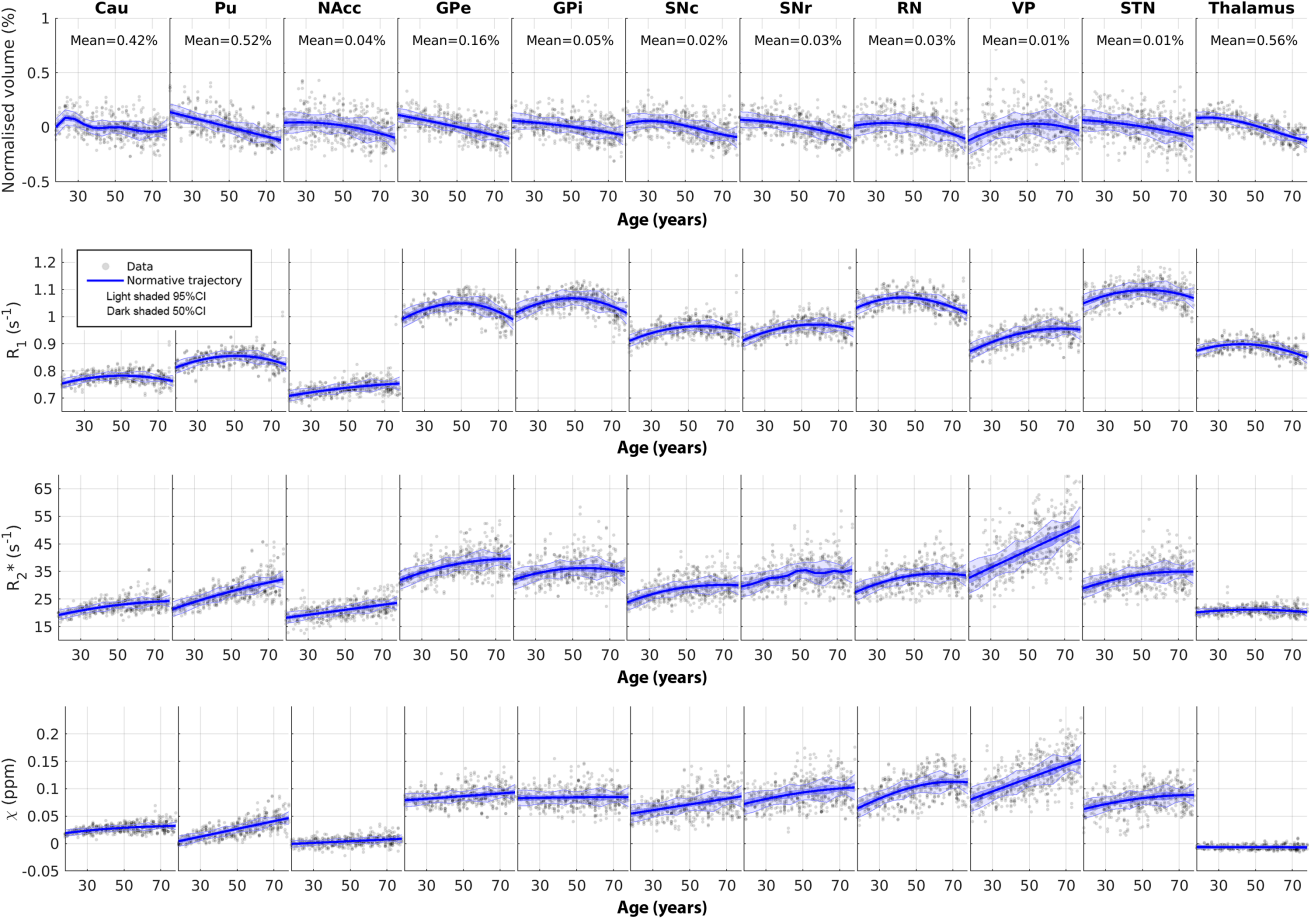
Normative models of normalised volume, R_1_, R_2_*, and*χ*as a function of age for 11 different deep grey matter structures present in the MuSus-100 atlas. For visualisation purposes, the normalised volume of a given structure,$V_{ROI,\%}$, is expressed as a percentage change of tissue volume inEquation (2)and is shown at the top of each plot. The data points shown on the scatter plot were corrected for sex and hemispheric effects. The blue shaded regions represent the 50% and 95% confidence intervals (CI) on the derived normative trajectory.

### 3.3 GPR normative trajectories of thalamic sub-nuclei

**Fig. 4.**
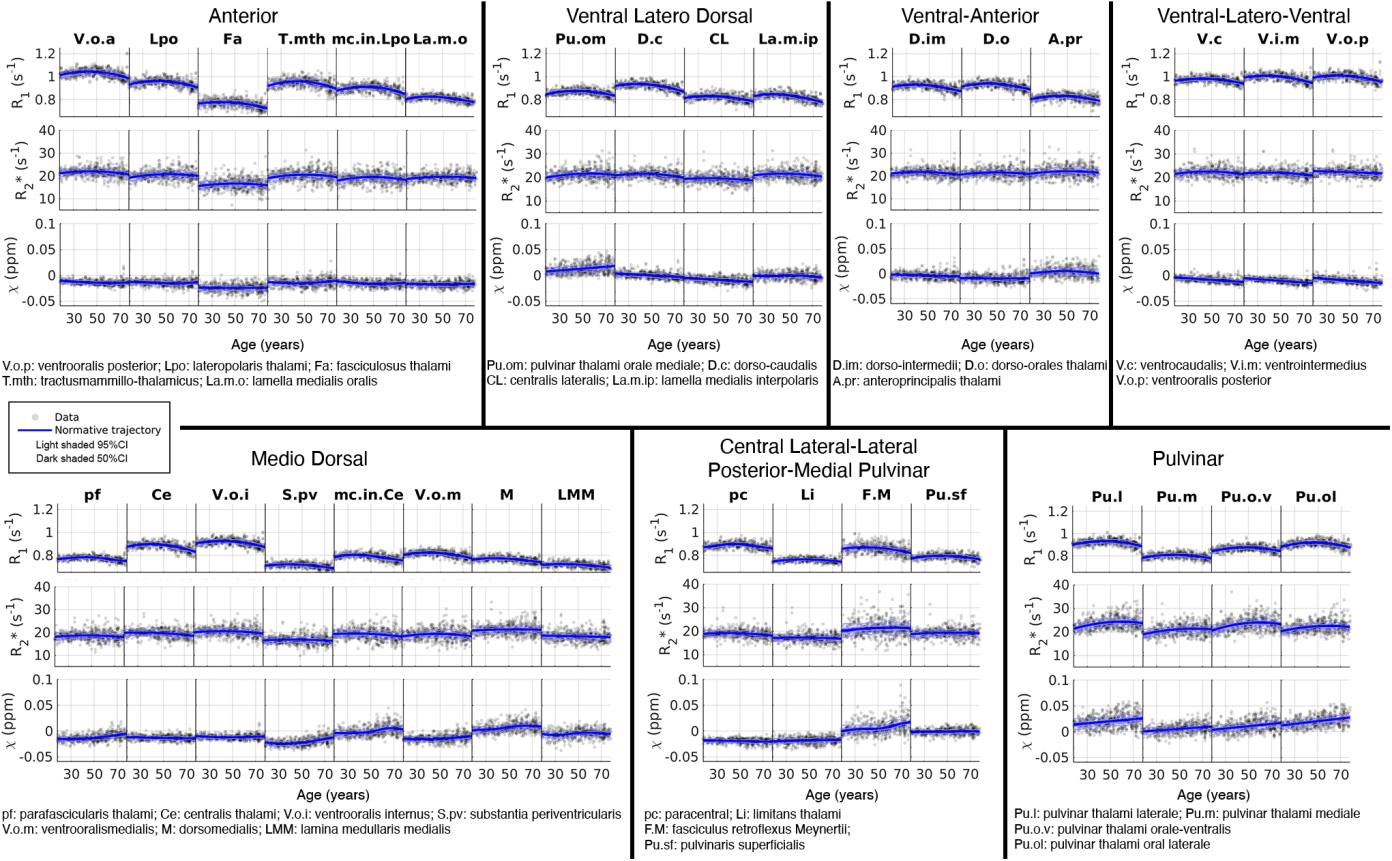
Normative models for the thalamic sub-nuclei defined in the MuSus-100 atlas as a function of age. To facilitate visualisation of the trajectories, we have clustered these trajectories based on their anatomical proximity to the seven thalamic sub-parts defined by the maximum likelihood atlas (Najdenovska et al., 2018).

The trajectory of*χ*was nearly constant when treating the thalamus as a single structure (bottom right,Fig. 3). However, further investigation of each thalamic nucleus revealed that the nuclei had distinct trajectories (seeFig. 4), and ageing showed opposite effects on*χ*in various structures. For example, the*χ*value increased as a function of age for nuclei close to the pulvinar region (bottom right,Fig. 4), while an opposite trend was observed in the ventral-latero dorsal, ventral-anterior, and ventral-latero-ventral ROIs (top row,Fig. 4). In contrast to*χ*, R_1_and R_2_* were relatively comparable among the thalamic nuclei although they clearly had different means across the cohort population, suggesting that an alternative clustering of thalamic nuclei could be obtained using mean R_1_and R_2_* values. Among the thalamic nuclei, the V.o.a (first column, top left,Fig. 4) and the Pu.I (first column, bottom right,Fig. 4) had the largest R_1_and R_2_*, respectively, and the S.pv (fourth column, bottom left,Fig. 4) and the Fa (third column, top left,Fig. 4) had the lowest R_1_and R_2_* values, respectively.

### 3.4 Spatial normative trajectories of putamen and caudate nucleus

**Fig. 5.**
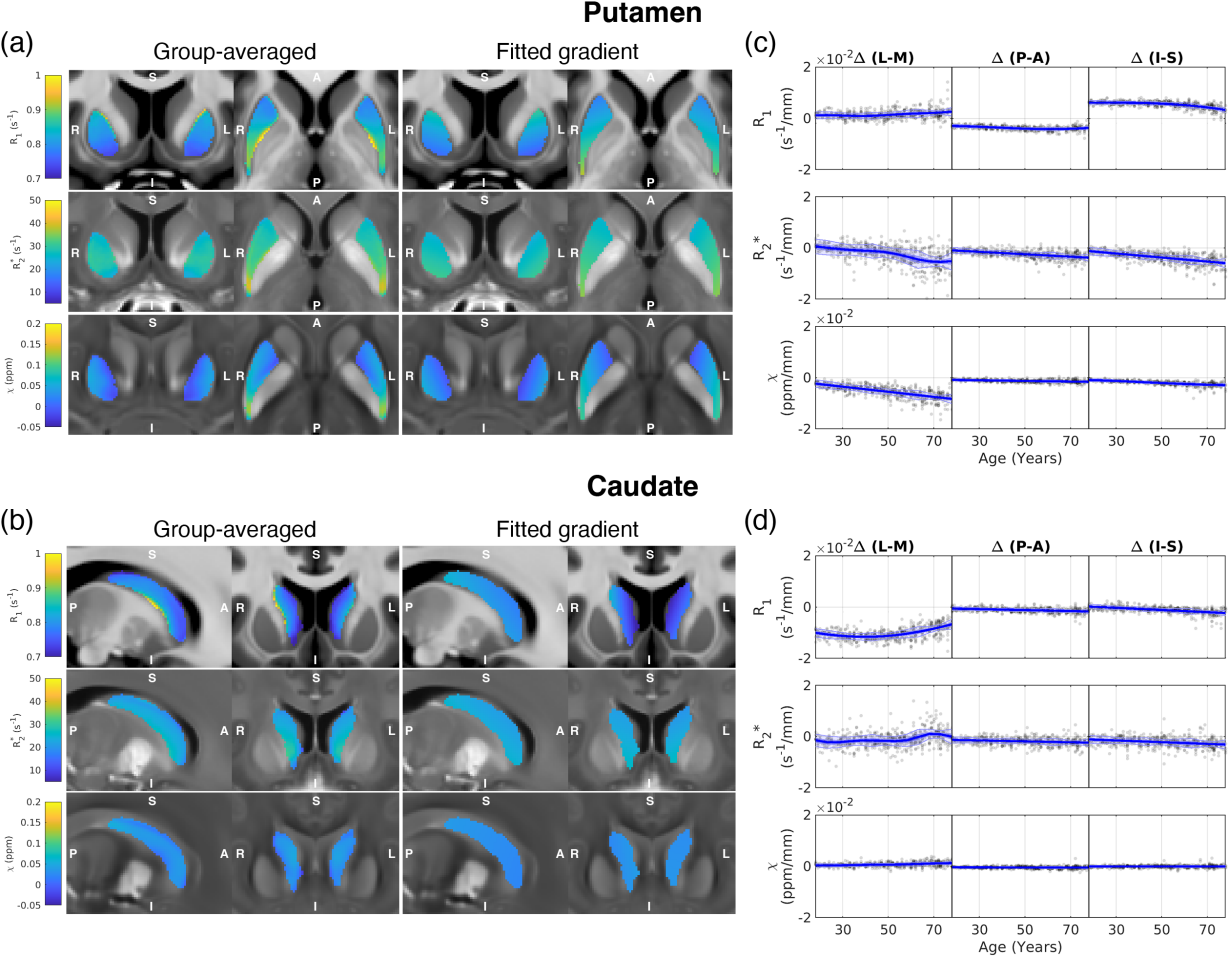
Example gradient overlaid on anatomical R_1_, R_2_*, and*χ*images in the (a) putamen and (b) caudate nucleus using group-averaged qMRI maps. The normative trajectories of the qMRI metrics in L-M, P-A, and I-S directions in the (c) putamen and (d) caudate nucleus.

Figure 5a and bshowcases the spatial variations on the group-averaged maps observed in the two structures, revealing a predominantly linear gradient. In the putamen, R_1_exhibited a non-zero gradient offset in the Posterior-Anterior (P-A) and Inferior-Superior (I-S) directions, indicating spatial gradients presented across all ages (top rowFig. 5c), and these were not associated with the presence of distinct nuclei as in the thalamus. The age-dependent effect on the spatial gradient was evident in the Lateral-Medial (L-M) direction for R_2_* and*χ*, with the magnitude of the gradient increasing from medial to lateral with age (first column ofFig. 5c). Similarly, age-dependent effects were observed in the P-A and I-S directions for R_2_*, albeit weaker than the L-M direction. In the caudate nucleus, a non-zero gradient offset was identified in the L-M direction for R_1_, with a parabolic change in gradient magnitude as a function of the subjects’ age (top corner,Fig. 5d).

### 3.5 Normative trajectories of basal ganglia beyond median qMRI and correlation analysis between qMRI metrics

**Fig. 6.**
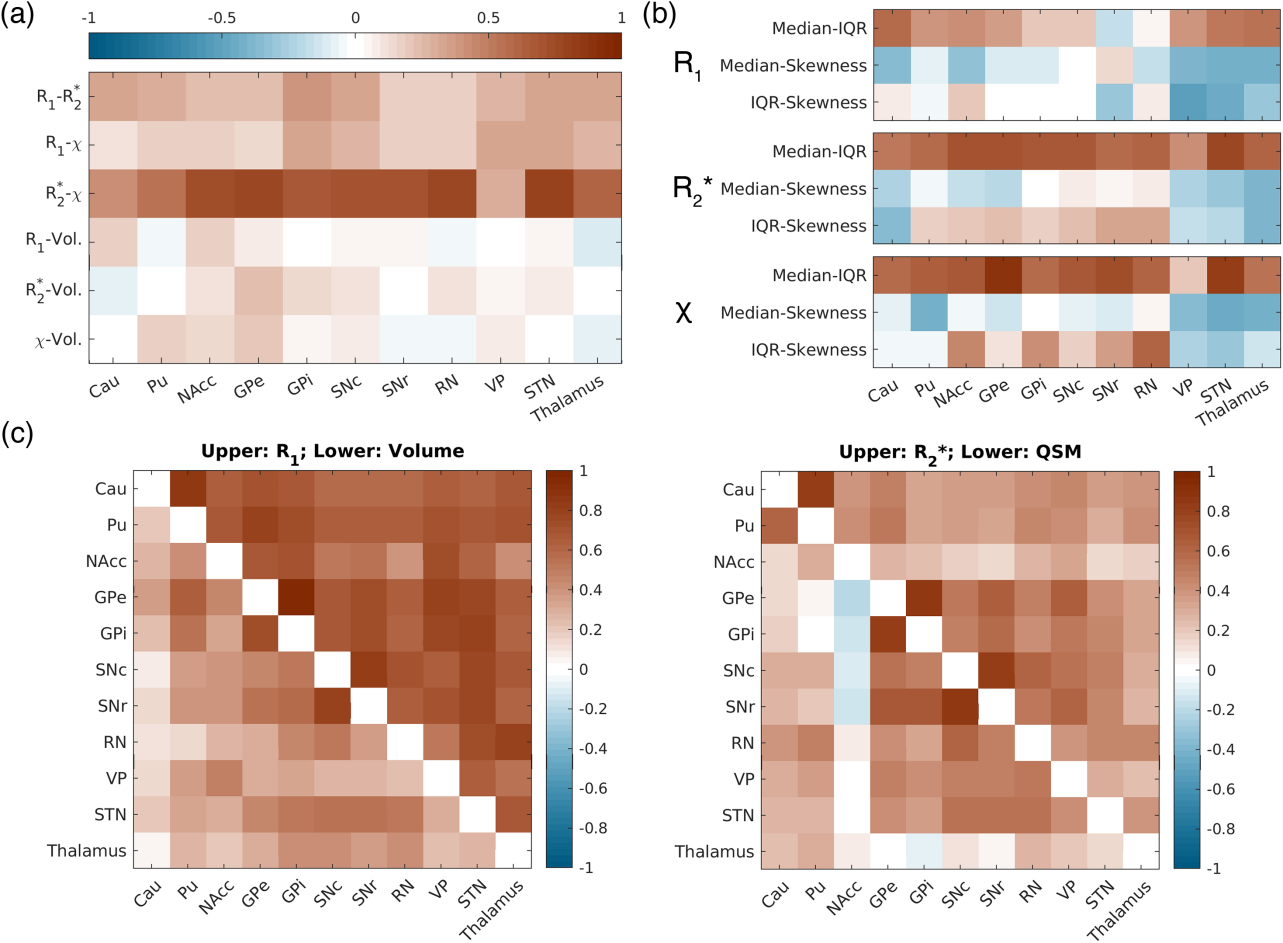
Pearson’s correlations between the z-scores derived from the normative models of both the volumetric and qMRI metrics: (a) correlation between the z-scores across the four different metrics derived fromFigure 4(volume, R_1_, R_2_*, and*χ*) per ROI; (b) correlations within each qMRI metric between the z-scores of all subjects between the three statistical measures (median, IQR, and skewness); (c) and (d) correlation matrices for volumetric and qMRI z-values across ROIs.

Weak-to-moderate correlations (0.17 ≥ R ≥ 0.39) were observed between R_1_and R_2_*, as well as between R_1_and*χ*(0.10 ≥ R ≥ 0.34) across all subcortical nuclei (Fig. 6a). Moderate-to-strong correlations (0.44 ≥ R ≥ 0.81) between R_2_* and*χ*were observed in all nuclei except VP, indicating a linear relationship between changes in R_2_* and*χ*(third row,Fig. 6a), after accounting for the strong effects of age. Conversely, changes in subcortical nucleus volume exhibited only weak correlations with their corresponding qMRI metrics (Fig. 6a).

We observed that robust normative trajectories can also be extracted from the IQR of each nucleus (Supplementary Fig. S7), while the nuclei exhibited different offsets in skewness and remained relatively invariant across age with relatively strong variances (Supplementary Fig. S7). When examining the correlation between different univariate analysis metrics (such as median, IQR, and skewness) of the same ROI and qMRI metric,Figure 6breveals a notable correlation between median values and IQR (first row), with the correlation values comparable with those found between R_2_* and*χ*inFigure 6a, whereas skewness demonstrated a reduced correlation with median and IQR, respectively.

Moving to the investigation of qMRI covariance networks, upon closer examination of individual metrics, R_1_displayed the strongest correlations in changes among the nuclei compared with other metrics (upper triangle, left panel,Fig. 6c). Particularly strong correlations were observed in R_1_between the caudate nucleus and putamen (R = 0.87), GPe and GPi (R = 0.95), and SNc and SNr (R = 0.84). In R_2_* and*χ*, two clusters of strong correlation were observed: one between the caudate nucleus and putamen (R = 0.81 for R_2_* and R = 0.61 for*χ*), and another among the globus pallidus, substantia nigra, red nucleus, and subthalamic nucleus (right panel,Fig. 6c). The correlation matrix on volume changes was overall less similar to other metrics (lower triangle, left panel,Fig. 6c), despite exhibiting the strongest correlation between GPe and GPi, and between SNc and SNr.

### 3.6 Validation of normative trajectories on the PPP dataset

**Fig. 7.**
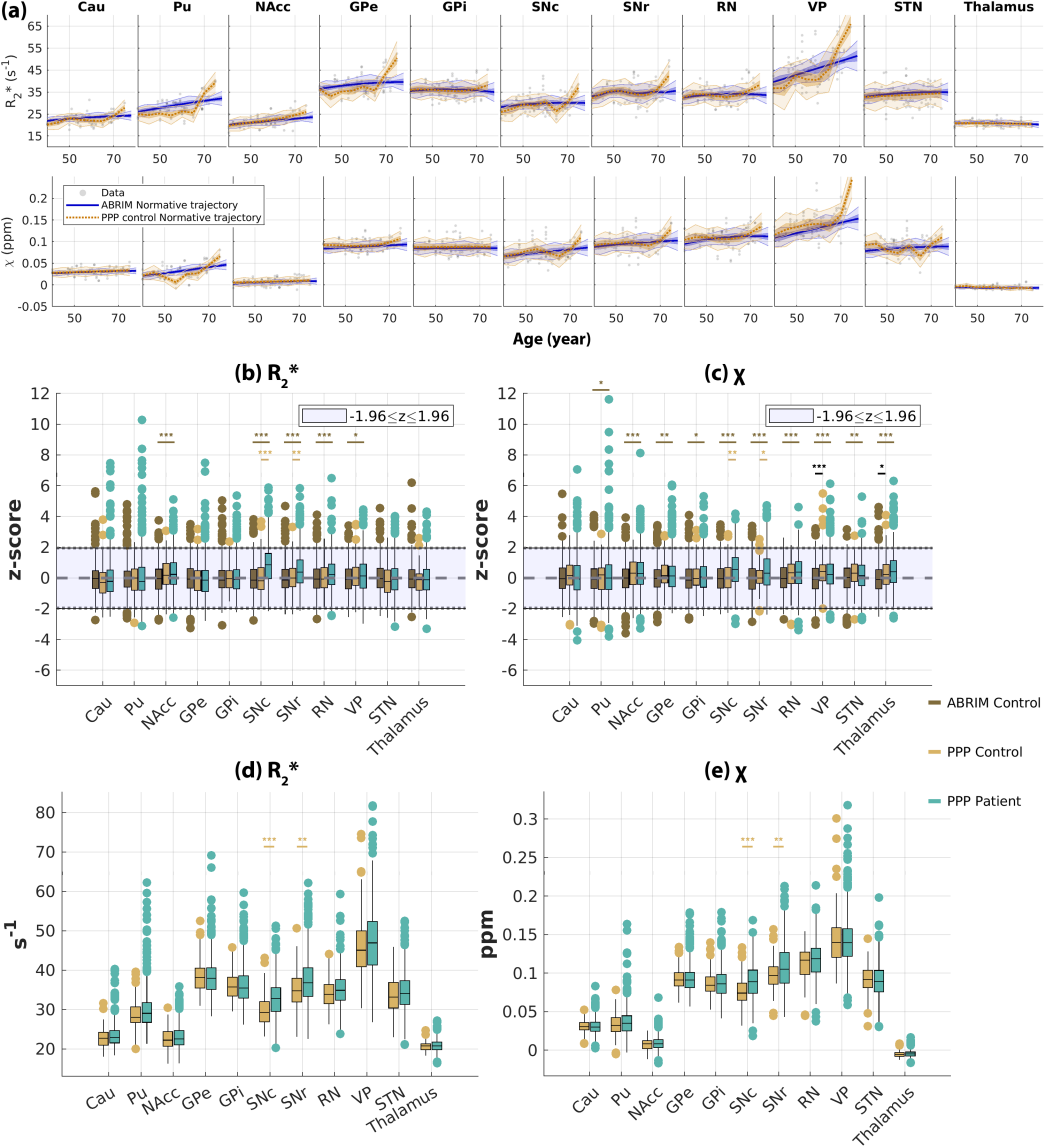
(a) Scatter plots of R_2_* (top row) and*χ*(bottom row) values as a function of age in an independent sample of healthy controls (N=44) obtained from the PPP (Johansson, Toni, et al., 2023). Blue and orange lines represent the normative models derived from the ABRIM study (as shown inFig. 3) and derived from the new control data. Panels (b) and (c) show the derived z-scores on the PPP and ABRIM data using the ABRIM normative models for R_2_* and*χ*, respectively. Panels (d) and (e) show the sex and interhemispheric-corrected qMRI values on the PPP data for R_2_* and*χ*, respectively. Within each panel, whisker plots are used to characterize the distribution (25, 50, and 75 percentile) of the controls (dark and light yellow) and Parkinson’s disease patients (green). *p-value < 0.05; **p-value < 0.01; ***p-value < 0.001.

Finally, we explored the applicability of the R_2_* and*χ*normative trajectories using another dataset comprising both healthy individuals and Parkinson’s disease patients, characterised by a narrower age range in this cohort. When plotting the healthy individual data together with the normative models shown inFigure 3, most of the data points visually aligned with the normative trajectories (Fig. 7a). Yet, the normative models derived from this narrower range of data (orange lines,Fig. 7a) were less smooth when compared with having the wider age range data (blue lines,Fig. 7a), particularly for the putamen, VP, and STN, suggesting the models may be overfitted and not account for the true age dependence. The robust fits of our normative models to the new data were highlighted by the distribution of the z-scores for all ROIs. The median z-scores were close to zero for almost all subcortical nuclei, and the z-score distributions of the PPP healthy controls fell within the range of |z|≤1.96 (~95% CI) for both R_2_* (light yellow boxes,Fig. 7b) and*χ*(light yellow boxes,Fig. 7c), though relatively stronger deviations were observed on R_2_* of the caudate nucleus, and*χ*of GPe, RN, and VP. When examining Parkinson’s disease patients, it is notable that most distributions remained centred around zeros, with exceptions noted for SNc and SNr (green boxes,Fig. 7b, c). Two-sample t-test results indicated that the z-scores of both R_2_* and*χ*in SNc were significantly higher in the Parkinson’s disease patients than in the healthy controls (adjusted*p*-values = 6.8 x 10^-7^and 1.5 x 10^-3^for R_2_* and*χ*, respectively) and in SNr (adjusted*p*-values = 5.8 x 10^-3^and 0.018 for R_2_* and*χ*, respectively). Similarly, these two structures also exhibited significant differences between the two groups when comparing the R_2_* and*χ*values directly (adjusted*p*-values of SNc = 2.6 x 10^-7^and 1.5 x 10^-4^for R_2_* and*χ*, respectively; adjusted*p*-values of SNr = 4.8 x 10^-3^and 6.6 x 10^-3^for R_2_* and*χ*, respectively;Fig. 7d, e). When comparing the Parkinson’s disease patients of the PPP dataset with the ABRIM dataset comprising more healthy control subjects, NAcc, SNc, SNr, and RN showed highly significant differences (adjusted*p*-value < 0.001) on both R_2_* and*χ*(dark yellow,Fig. 7b, c). Additionally, VP and thalamus also showed highly significant differences on*χ*(adjusted*p*-value < 0.001) between the two populations. In contrast, only VP showed a highly significant difference in*χ*when comparing the ABRIM population with the PPP healthy control group.

## 4 Discussion

In this work, we demonstrated the effectiveness of integrating normative modelling with qMRI techniques at 3T. We believe that this study represented the most comprehensive single-study investigation to date of the variations in the three quantitative metrics (R_1_, R_2_*, and*χ*) in the basal ganglia throughout adulthood. Our study benefited from a large sample size of 260 healthy participants, with a balanced distribution of age and sex. This allowed us to explore not only the evolution of these metrics with age beyond the simple young versus old adult comparison but also their heteroscedasticity, that is, how the variance changes across different age groups. Prior studies focusing on R_1_, R_2_*, and*χ*have primarily utilised 7T data and typically involved smaller sample sizes (Keuken et al., 2017). While larger studies at 3T do exist, they have mainly focused on R_2_* and*χ*(Y. Li et al., 2021;Treit et al., 2021). Our study provides additional comprehensive resources to the existing literature in understanding how brain development during adulthood impacts multiple qMRI parameters, including R_1_, R_2_*, and*χ*.

The inverted U-shaped trajectory observed in R_1_has been in the past linked to processes of myelination and demyelination when investigating white matter (Yeatman et al., 2014). While refraining from overinterpreting the significance of R_1_and its association with macromolecular content or myelination, it is noteworthy that the peak age in our normative trajectories typically falls between 50 and 65 years, considerably later than the observations in white matter bundles (Yeatman et al., 2014). One explanation for this shift in the maturation peak measured in R_1_could be attributed to the overlap of a myelination trajectory that peaks at an earlier age, with a linear increase of iron deposition. Such a shift would not be observed in R_1_of white matter where iron concentrations are orders of magnitude lower. Similar age trends were generally observable in R_1_across subcortical nuclei, and to a lesser extent in R_2_* (as evidenced by the differences in curvatures in R_1_and R_2_* inFig. 3), while*χ*appeared to be less influenced by the myelination process in subcortical nuclei. It is important to note that*χ*has been identified as highly sensitive to myelination in white matter (Liu et al., 2011;Lodygensky et al., 2012). This sensitivity is primarily attributed to microstructural effects that are amplified in tightly aligned white matter bundles (Luo et al., 2014;Wharton & Bowtell, 2015), which are less prevalent in subcortical nuclei.

It is well established that R_2_* and*χ*encode similar contrast mechanisms albeit in different ways (Stüber et al., 2014), and they are particularly sensitive to iron concentration when examining subcortical nuclei (Langkammer et al., 2012). This similarity is underscored by the strong resemblance of the normative models depicted inFigure 3(3^rd^and 4^th^rows). There was a discernible association between regions with higher and lower values and the rates at which they changed throughout adulthood. Even after removing the age confound and transforming the metrics of interest to z-statistics, our correlation analysis depicted inFigure 6aindicated a high level of interrelation between these two parameters (mean correlation R_2_* vs.*χ*= 0.64). Although the correlation of these values to the z-scores of R_1_, which is believed to be predominantly associated with MT effects, was diminished (mean correlations of R_1_vs. R_2_*=0.28; R_1_vs.*χ*= 0.23), it confirmed that iron contributes weakly to the contrast observed in subcortical nuclei as previously shown (Rooney et al., 2007). InSupplementary Figures S7to S8 andFigure 6b, we have demonstrated those statistical measures beyond the median and the spatial gradients, such as skewness and IQR. We observed positive correlations between median and IQR in most of the ROIs (Fig. 6b), suggesting the spread across an ROI becomes greater with the increase in measured values. It is not possible to disentangle the source of these correlations: estimation precision will affect both IQR and will be reduced as the median values increase (e.g., higher R_2_* will result in reduced SNR); spatial variation within ROI can contribute to IQR (as also mapped by the gradients, inFigure 5, and the coefficient of variation in the template inSupplementary Fig. S3) and are more likely to occur when there is an increase in the underlying contrast mechanism (and thus increase of the median value). Overall, these measures were relatively noisier than the median. Yet, they have the potential to serve as additional imaging biomarkers when combined with local spatial information (G. Li et al., 2019). Volumetry is frequently utilised in ageing and cohort studies (Bethlehem et al., 2022;Rutherford, Fraza, et al., 2022), yet our results suggest that the process of volume change is largely distinct from the microstructural alterations (including macromolecular concentration and iron deposition) measured by the three qMRI methods addressed herein. Future studies to explore the association between qMRI metrics and behavioural data available in the ABRIM data collection (Jansen et al., 2024) may provide valuable insight into cognitive ageing with (micro)structural alterations.

Our examination of the normative trajectories of various thalamic nuclei, as depicted inFigure 4, yielded an unexpected observation. We found that while the trajectories of R_2_* either remained constant or exhibited minor increases, the*χ*values showed small but noticeable increases or decreases with age, varying across different nuclei. One possible explanation for these changes, beyond the susceptibility changes resulting from the actual structural alteration, could be related to arbitrary susceptibility reference used in the QSM processing (QSM Consensus Organization Committee et al., 2024). If the mean of the absolute magnetic susceptibility of the whole brain would be increasing at a higher rate than that of those thalamic nuclei (D.c, CL, V.c, V.i.m, and V.o.p.), then our assumption that the mean brain susceptibility of the brain remains equal to zero would lead us to expect an apparent decrease of the measured relative susceptibility.

Spatial variation in R_1_and*χ*within the putamen and caudate nucleus has been previously documented by other research teams, with variation observed between young and elderly individuals in both R_1_and*χ*(Keuken et al., 2017). This spatial gradient may hold significance in the context of Parkinson’s disease (Keuken et al., 2017), as alterations in the anterior-posterior (A-P) gradient in the R_1_maps have been observed in Parkinson’s disease patients compared with age-matched healthy individuals (Drori et al., 2022). Furthermore, the tails of the putamen in*χ*maps have been found to be significantly associated with decreased motor performance in Parkinson’s disease patients (Drori et al., 2022). Extending this analysis to other subcortical nuclei may provide further insights into age-related maturation that are more specific to localised spatial changes, though higher spatial resolution data might be required to accurately capture the spatial variation for small structures. The thalamus could be another structure of interest to study the changes in spatial variation across age, and previous studies have demonstrated gradient changes of MR properties from outer to inner thalamic layers in multiple sclerosis (Rubin et al., 2024). Nonetheless, careful consideration of how to classify the thalamus into different subparts is needed since the thalamic nuclei have various developmental trajectories and median values as demonstrated inFigure 4.

Here, we opted to conduct normative modelling using high SNR approaches derived from atlas-based parcellation. The atlas used here (MuSus-100) was constructed using similar qMRI maps. To optimise the coregistration between our dataset and the associated templates from the atlases, we implemented a robust method to address the image contrast variations between site-specific data and the atlas template. We also extended our analysis to another atlas (Alkemade et al., 2020) to demonstrate the robustness of this approach for subcortical parcellation (seeSupplementary Fig. S6). The contrast variations in the hybrid image may arise due to differences in MRI scanner field strength, imaging sequences, or the signal model used to derive the qMRI values, potentially introducing image intensity biases between datasets. To streamline the parcellation process based on volumetric image registration, we integrated Step 1 of the co-registration procedure into the SEPIA toolbox (Chan & Marques, 2021), aiming to facilitate the widespread adoption of this methodology. Accurate image registration is crucial for atlas-based parcellation, but the quality of registration can still vary between subjects due to differences in individual morphology and MR properties, even with meticulous processing. Minor registration inaccuracies, particularly near the boundary of parcellation labels, are not uncommon (e.g., seeSupplementary Fig. S4). To reduce the partial volume effect caused by these inaccuracies, we used the median rather than the mean when deriving the normative trajectories, as the median is more resistant to outliers. However, the partial volume effect can still impact the robustness of the derived models, particularly in thalamic nuclei, due to their relatively small size and the less distinct image contrast with the surrounding tissue.

While our focus has been on examining normative models for individual ROIs, it is essential to acknowledge that this information is not entirely independent across the brain. We observed correlation values exceeding 0.5 for almost all ROIs for R_1_. This could either indicate a potential consistent measurement bias, such as inaccurate B_1_compensation during R_1_computation or suggest that there is a common macromolecular change influencing all brain regions similarly due to co-development or co-regulation (Carmon et al., 2020). Despite our careful consideration of the B_1_effect (Marques & Gruetter, 2013), there may still be residual biases due to differences in inversion efficiency among subjects, where the system might have underestimated or overestimated the reference voltage. For the R_2_* and*χ*z-scores, strong correlations were observed between the caudate nucleus and putamen, as well as among the sub-structures of the globus pallidus and substantia nigra (R >0.6), possibly driven by their close spatial proximity (Fig. 6c). By reducing the correlation threshold to 0.4, we can visually discern two main “networks of variations”: one comprising the caudate nucleus and putamen, and the other including the globus pallidus, substantia nigra, red nucleus, ventral pallidum, and subthalamic nucleus, indicating potentially there are two mechanistically different processes of iron deposition in the brain.

When we applied the normative models to study the R_2_* and*χ*differences between healthy controls and Parkinson’s disease patients, we were able to reproduce previously reported group differences in both substantia nigra subregions, where significant increases of R_2_* and susceptibility values were observed (Biondetti et al., 2021;Mitchell et al., 2021), particularly in SNc (Fig. 7b, c). In contrast to comparing the median R_2_* and*χ*values between the two groups (Fig. 7d, e), the*p*-values in the z-score comparison were higher in the substantia nigra. One possible explanation is that although the two groups had a similar mean and SD of age, there were still differences in the age distributions between the two groups (seeSupplementary Fig. S1), which would introduce a bias when comparing the two groups using the quantitative MRI values uncorrected for age. It is also possible that we may not have sufficient data in the ABRIM dataset to accurately capture the true variance for all ages using the Gaussian Process Regression in the normative modelling (as suggested by the zig-zag pattern of the 95% CI between successive ages inFig. 3). Methods such as Bayesian linear regression (Rutherford et al., 2023) or hierarchical Bayesian regression (de Boer et al., 2024) combined with cubic beta spline basis sets with a reduced number of knots should also be evaluated in future studies to find a better compromise between normative trajectory flexibility and robustness to data outliers. Finally, we evaluated the use of the ABRIM dataset as our control cohort (despite the acquisition and segmentation protocols not being exactly matched, see methods section). The increase in control population size resulted in more regions with highly significant differences (*p*< 0.001) between patients and controls, from 1 to 4 in R_2_* (NAcc, SNr, SNc, and RN) and from 0 to 6 in*χ*(NAcc, SNr, SNc, RN, VP, and thalamus). While a study-associated bias might also be attributed to this observation, when comparing the two control populations, only*χ*of the PPP control group was statistically higher than that of the ABRIM population in VP (*p*< 0.001) and thalamus (*p*< 0.05).

We emphasise the significance of creating normative models for each quantitative metric and ROI to understand these metrics within the context of a population, it is also important to recognise that these derived models, even within the realm of quantitative imaging, may not hold universal validity across different studies. Variations in acquisition parameters, including repetition times, echo times, inversion times, image resolution, and vendor hardware, as well as differences in the exact pipeline implementation to derive ROI metrics, are bound to introduce study biases (Karakuzu et al., 2022;Rowley et al., 2024;Stikov et al., 2015;Teixeira et al., 2020;Tsialios et al., 2017). Therefore, when combining datasets from different sites, it is crucial to consider harmonisation procedures akin to those commonly employed in other morphometric analyses to account for site effects (Bayer et al., 2022;Fortin et al., 2017;Kia et al., 2020;Pomponio et al., 2020). In this work, we pooled the subcortical nuclei from the left and right hemispheres together to study their normative trajectories across ages. However, the development/maturation between the left and right hemispheres may not be the same, even for the same structure. We utilised the GLM model to account for the inter-hemispherical difference in this work. Yet, for future studies with more data available, the normative trajectories of each structure from different hemispheres could be studied separately, which would allow us to explore the inter-hemispheric differences in brain development more accurately. Both the raw quantitative maps and the derived quantitative metrics from our healthy volunteers, encompassing various subcortical nuclei and atlases evaluated here, have been made publicly available to facilitate integration into subsequent research endeavours (Jansen et al., 2024).

## 5 Conclusion

In this study, we conducted a comprehensive normative modelling analysis of R_1_, R_2_*, and*χ*in subcortical nuclei and thalamic sub-nuclei across adulthood. Our analysis utilised a cohort with an evenly distributed age and sex ratio. The normative models we derived take into account the changes in variance across age, thereby enabling the derivation of z-statistics with new data. Interestingly, the shapes of the trajectories align with existing literature findings despite our use of a non-parametric approach to compute these models. The observed differences in the trajectory shapes between R_1_and R_2_*/*χ*suggest that the mechanisms driving these contrasts are not identical. We further confirmed that the putamen and caudate nucleus exhibited an age-dependent spatial distribution of these qMRI metrics. This finding could potentially pave the way for more in-depth studies of spatial distribution changes in neurological disorders, such as Parkinson’s disease.

## Data and Code Availability

The ABRIM dataset and processing pipeline scripts that support the findings of this study are available athttps://doi.org/10.34973/atyn-r066. The processing pipeline scripts associated with the PPP dataset are available on the same repository but a separate agreement is needed to access the data:https://www.personalizedparkinsonproject.com/home/data/requesting.

## Author Contributions

Kwok-Shing Chan: Conceptualization, Methodology, Software, Validation, Formal analysis, Investigation, Writing—Original Draft, Visualization. Marcel P. Zwiers: Software, Resources, Data Curation, Writing—Review & Editing. Michelle G. Jansen and Martin E. Johansson: Data Curation, Writing—Review & Editing. Rick C. Helmich and Joukje M. Oosterman: Project administration, Funding acquisition, Writing—Review & Editing. David G. Norris: Supervision, Project administration, Funding acquisition, Writing—Review & Editing. Christian F. Beckmann: Conceptualization, Supervision, Funding acquisition. José P. Marques: Conceptualization, Methodology, Supervision, Writing—Original Draft, Visualization.

## Funding

The ABRIM study was funded by the European FP7 program, FP7-PEOPLE-2013-ITN, Marie-Curie Action, “Initial Training Networks” named “Advanced Brain Imaging with MRI” (no. 608123). CFB acknowledges funding from the Wellcome Trust Collaborative Award in Science 215573/Z/19/Z and the Netherlands Organization for Scientiﬁc Research Vici Grant No. 17854. The Personalized Parkinson Project (PPP) was co-funded by Verily Life Sciences LLC, the city of Nijmegen and the Province of Gelderland, Radboud University Medical Centre and Radboud University, as well The Michael J. Fox Foundation for Parkinson’s Research (grant ID #15581 to RCH). Allowance made available by Health~Holland (Top Sector Life Sciences and Health) to stimulate public–private partnerships. The Centre of Expertise for Parkinson & Movement Disorders was supported by a centre of excellence grant of the Parkinson’s Foundation.

## Declaration of Competing Interest

The authors have no competing interests related to the findings of this work.

## Supplementary Material

Supplementary Material
