# Supplementary material for: Normative trajectories of R 1 , R 2 *, and magnetic susceptibility in basal ganglia on healthy ageing
Source: Imaging Neurosci (Camb). 2025 Feb 3;3:imag_a_00456. doi: 10.1162/imag_a_00456 (PMC12319813; doi:10.1162/imag_a_00456)
Supplement: Supplementary Material [file imag_a_00456-supp.pdf]

Supplementary Materials

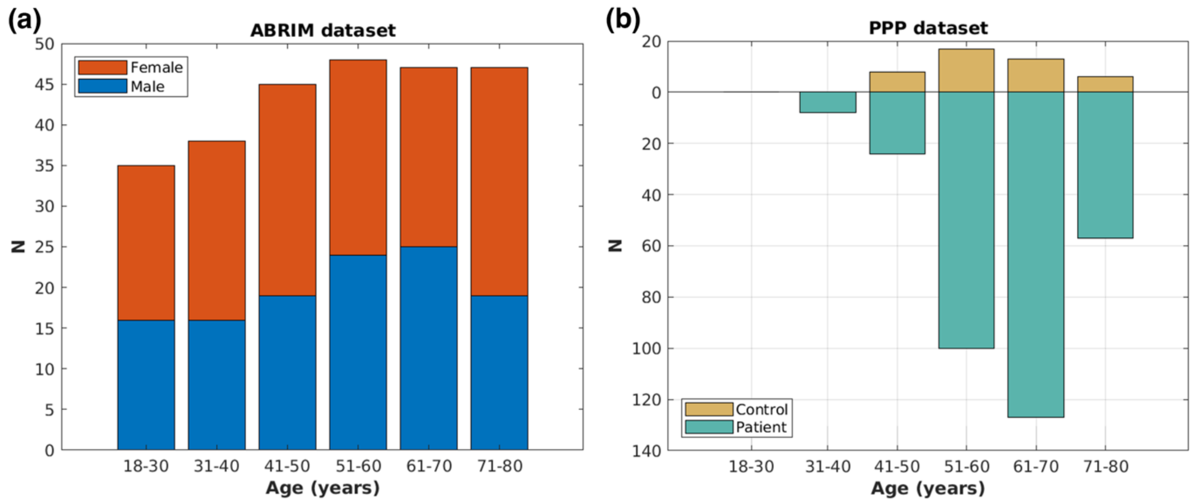

Figure S1: (a) Distribution of participants across the lifespan in the ABRIM dataset and (b) the PPP dataset.

Data Curation

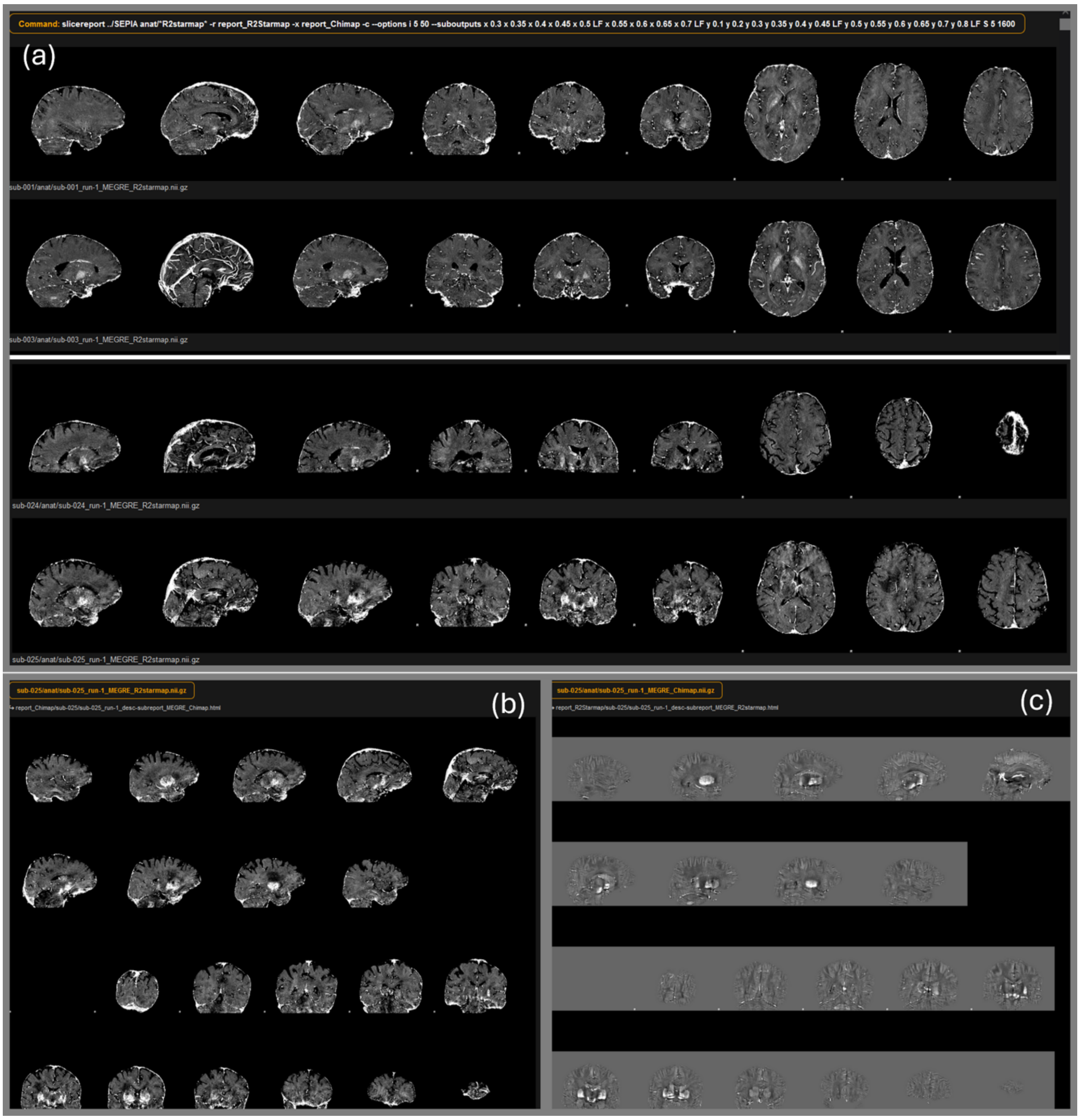

Figure S2: (a) Screenshot of the slice report of the R_2* maps HTML generated for the whole ABRIM study. The command on the top of the figure automatically generates an HTML report with all the R2starmap NIFTI files organised in a BIDS folder. The main study HTML page shows one subject per row, with 3 sagittal, 3 coronal and 3 axial slices at the centre of the brain using a predefined intensity range (5-50 s^{-1} in the case above). If the rater clicks on one of the images, he/she will automatically be directed to a subject-specific HTML page where a larger number of slices can be rapidly viewed. (b) shows the subject-specific R_2* map where a larger number of slices are shown. Additionally, it is possible to directly navigate to the subject-specific χ report (c), to quickly check if artefacts are also present on the corresponding susceptibility map.

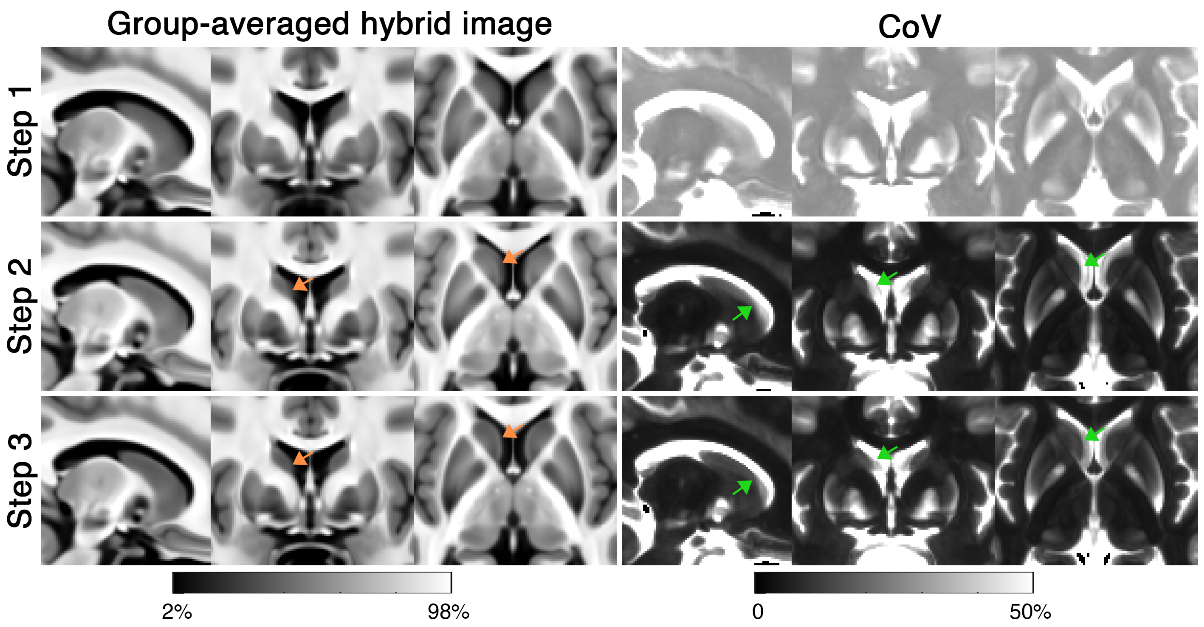

Figure S3: (left) Group-averaged hybrid image derived using the results of each registration step and (right) the corresponding coefficient of variation (CoV) image across all subjects. The result of Step 1 shows that the contrast between the globus pallidus and putamen is low without matching the contrast as in Step 2, resulting in blurry edges between the two close proximal structures. Note the boundary between the caudate nucleus and ventricle is notably sharper in the hybrid image of Step 3 compared to Step 2 (orange arrows). The CoV images also show lower values in the caudate nucleus/ventricle boundary at Step 3 (green arrows).

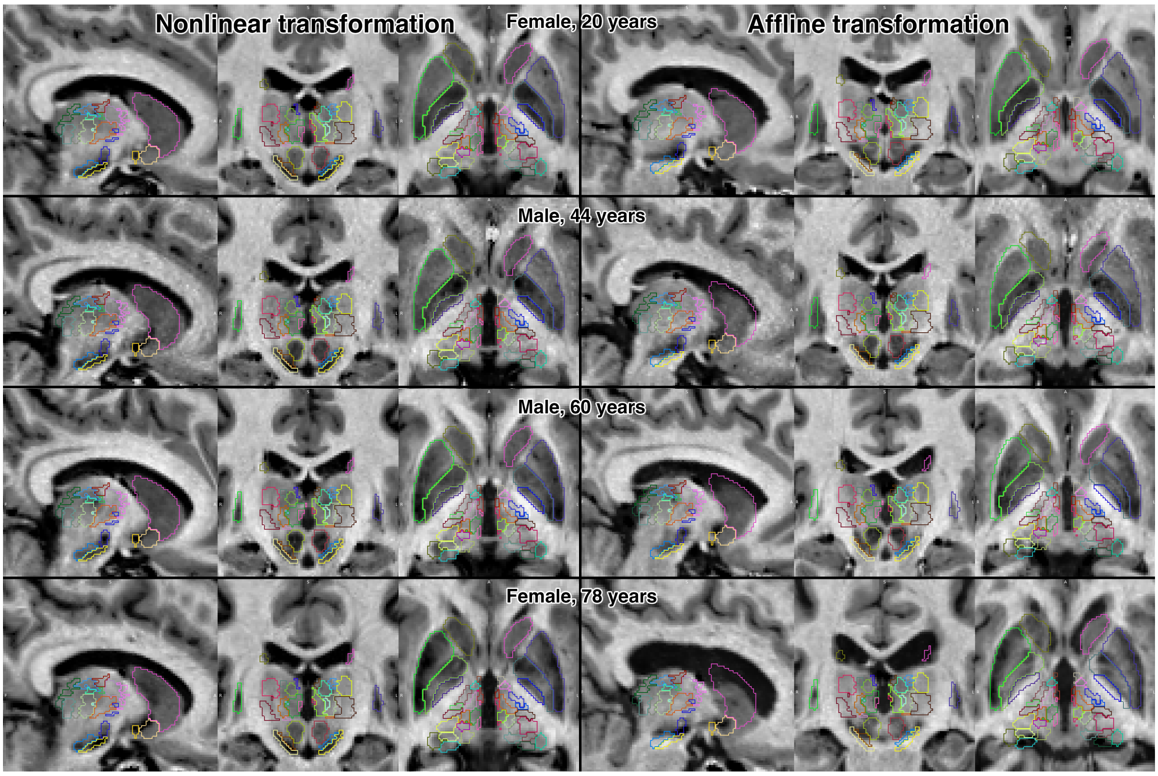

Figure S4: Examples of subcortical grey matter parcellation on 4 subjects of different ages. The MuSus-100 labels are overlaid (coloured outlines) on the subject hybrid image registered to the common study space using (left) nonlinear transformation and (right) affine transformation only.

Comparison of conventional MP2RAGE lookup table T_1 mapping and MP2RAGE dictionary matching

The conventional MP2RAGE T_1 mapping strategy is based on the use of a lookup table that relates the MP2RAGE combined image (which has a dynamic range from -0.5 to 0.5) and a T_1 value (Marques et al., 2010). While this approach is very time-efficient and even straightforward to combine with additional transmit B_1 maps to reduce residual B_1 sensitivity (Marques and Gruetter, 2013) it has some caveats. Most noticeably, the ability to map T_1 values is only guaranteed in a given range of T_1s where it is assumed that there is a non-bijective relationship between the combined MP2RAGE value and a given T_1. This range is controlled by the inversion times, their flip angles and TRs. As normally the T_1 of CSF is of limited interest, for the sake of efficiency, the range in which T_1 is correctly measured is limited to the range of T_1s of brain tissues. Although not reported in the original manuscript, it is not surprising that also an M_0 can be derived from the MP2RAGE sequence by simply dividing the signal intensity measured at the second inversion time, by the expected signal given the computed T_1. An alternative way to perform T_1 and M_0 mapping is fitting the 2 data points by the signal model (a two-variable fit to a two-point measurement). A computationally simple form to operationalise this is via a dot product between the measured signal (once the polarity has been recovered) and a dictionary of signal intensity based on forward model signal simulations.

Both methods described above can account for transmit B_1 inhomogeneity as independently mapped (see https://github.com/Donders-Institute/MP2RAGE-related-scripts T1B1correctpackageTFL.m and MP2RAGE_dictionaryMatching.m). Below is an example based on a random/representative selected subject from the ABRIM study is given (Jansen et al., 2024) with the following sequence parameters for mapping purposes ($TR/TI_1/TI_2$= 6000/700/2400 ms, α_1= α_2=6°, TR_{FLASH}=6 ms, $N_{shots\ per\ readout}$ = 176). The figure below shows the derived M_0 and T_1 maps of each of the methods. It is clear when observing the M_0 maps

using the conventional MP2RAGE map (Fig. S6d) that the CSF value (which is close to 100% water) is underestimated and lower than what is estimated for white matter and grey matter, whereas it is not the case in the dictionary matching.

Using FSL's *BET* (Jenkinson et al., 2012), the brain was extracted using the second inversion time image. The dictionary matching of M_0 and T_1 values was used the empirically define regions of high SNR outside the brain (M_0>2000, blue in segmented insert of Figure S6f) and define CSF regions inside the brain (T_1>3200 ms, green). The CSF mask was then dilated (inside the BET-derived brain mask) using Matlab's function *imdilate* with a 3x3x3 sphere structure element to define regions likely to suffer from partial volume artefacts (orange). Finally, the regions inside the brain that were neither CSF nor in the interface ROI were defined as brain tissue (dark red).

When carefully analysing the scatter plot in Figure S6f, it is clear that the estimated brain tissue values are unchanged, but that dictionary matching seems to be able to better compute T_1 in the CSF area (where the T_1 is known to be approximately ~4000 ms) and in regions of affected by the partial volume effect. Outside the brain, the main difference arises in fat regions with very low T_1 values where the measurement also clearly fails in the shown example (see Figure S6b,d).

For the current study, the choice of the T_1 mapping approach will only result in minor differences, as most of the structures analysed do not share a boundary with CSF (except Caudate and Thalamus). The choice of the approach might be more relevant when studying cortical grey matter (Chen et al., 2024).

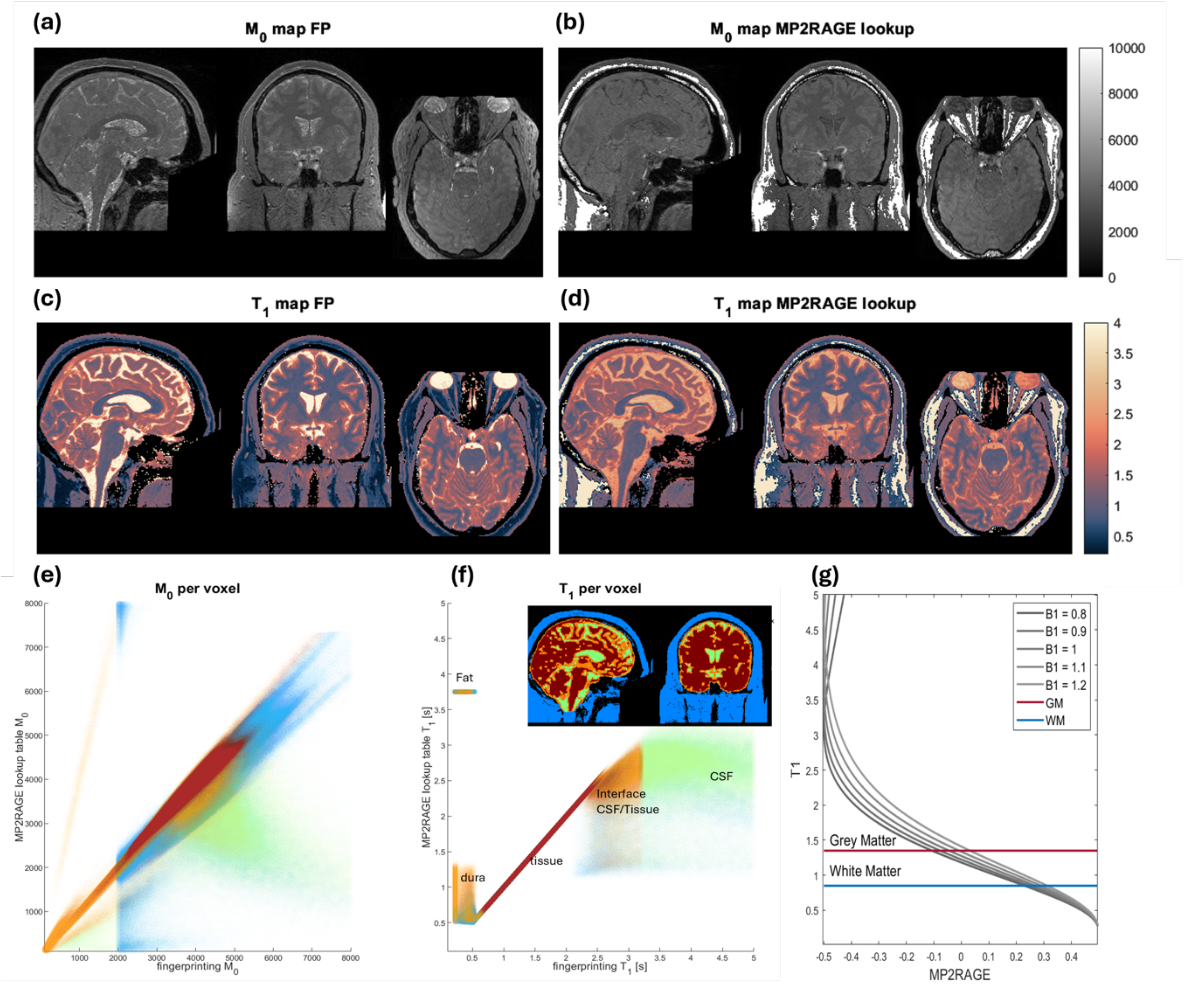

Figure S5: M_0 and T_1 maps derived by the dictionary matching (fingerprinting) strategy (a, c) and the conventional MP2RAGE look-up table (b, d), respectively. Panels (e) and (f) show the scatter plots of the derived M_0 and T_1 values respectively using the look-up table vs dictionary matching. Colours in scatter plots correspond to the classes shown in the inset segmentation. Panel (g) shows the lookup table of the ABRIM protocol for different B_1 fields.

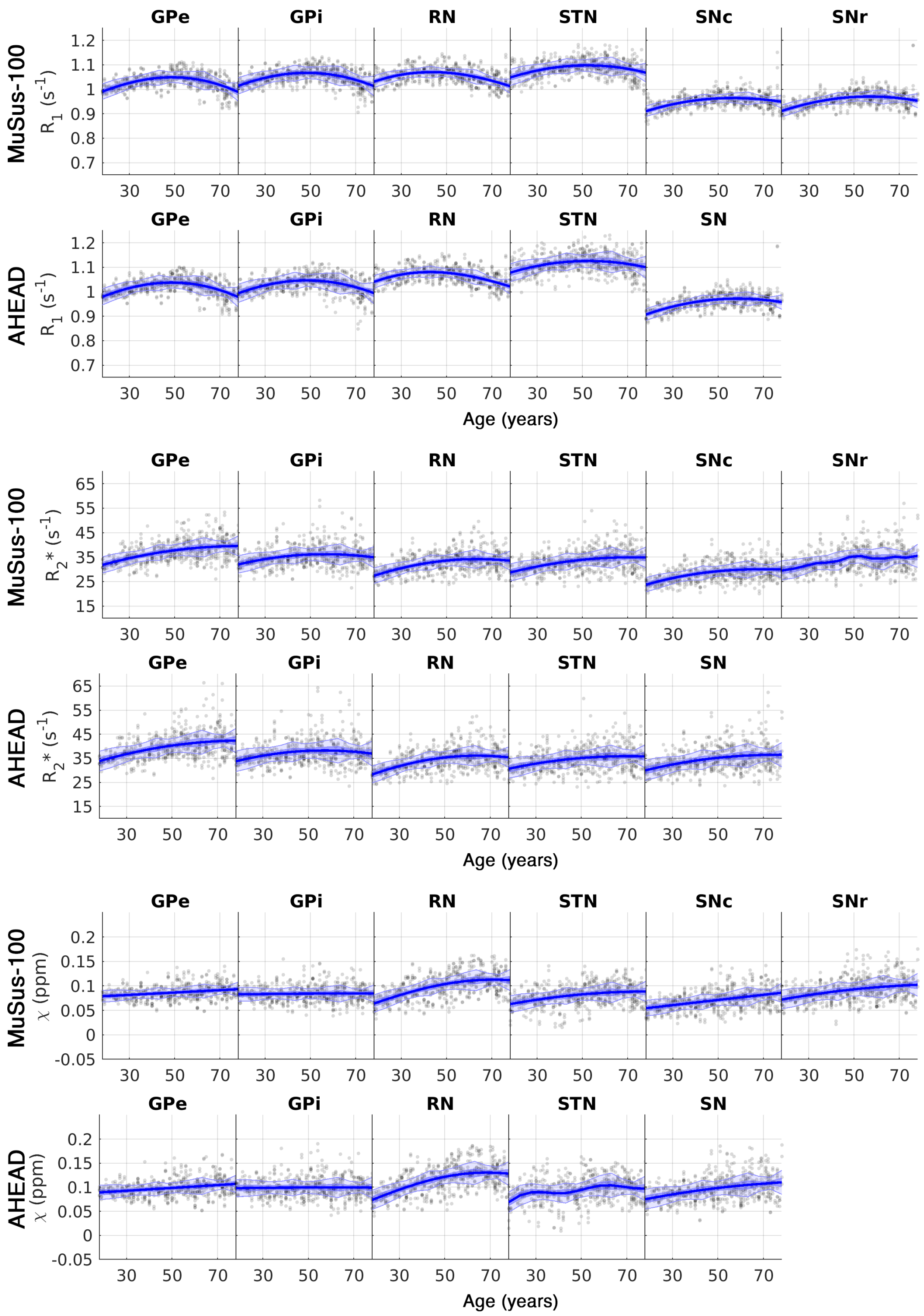

Figure S6: Normative trajectories derived using the AHEAD atlas (Alkemade et al., 2020) following the same process as described in the Methods section. The basal ganglia labels were obtained by hard thresholding the probabilistic images at 75%. The trajectories of the same structure derived using the MuSus-100 atlas are displayed alongside the AHEAD atlas results for direct comparison.

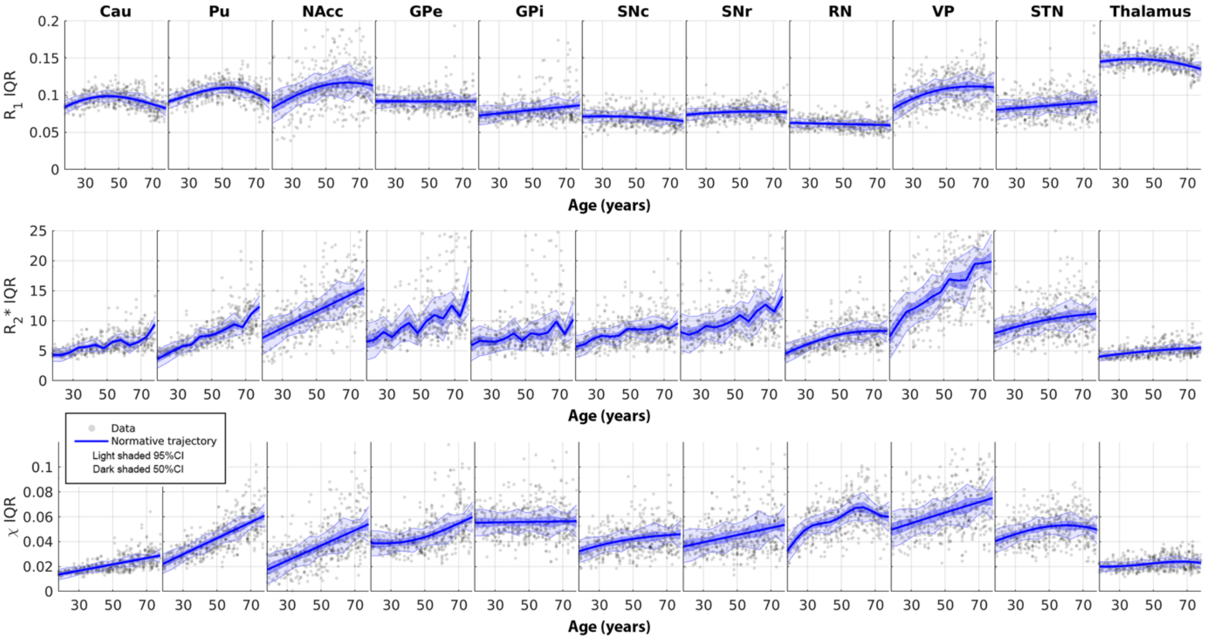

Figure S7: Normative trajectories of the interquartile range (IQR) of R_1, R_2^*, χ as a function of age for 11 different deep grey matter structures present in the MuSus-100 atlas. The data points shown on the scatter plot are corrected for sex and hemispheric effects.

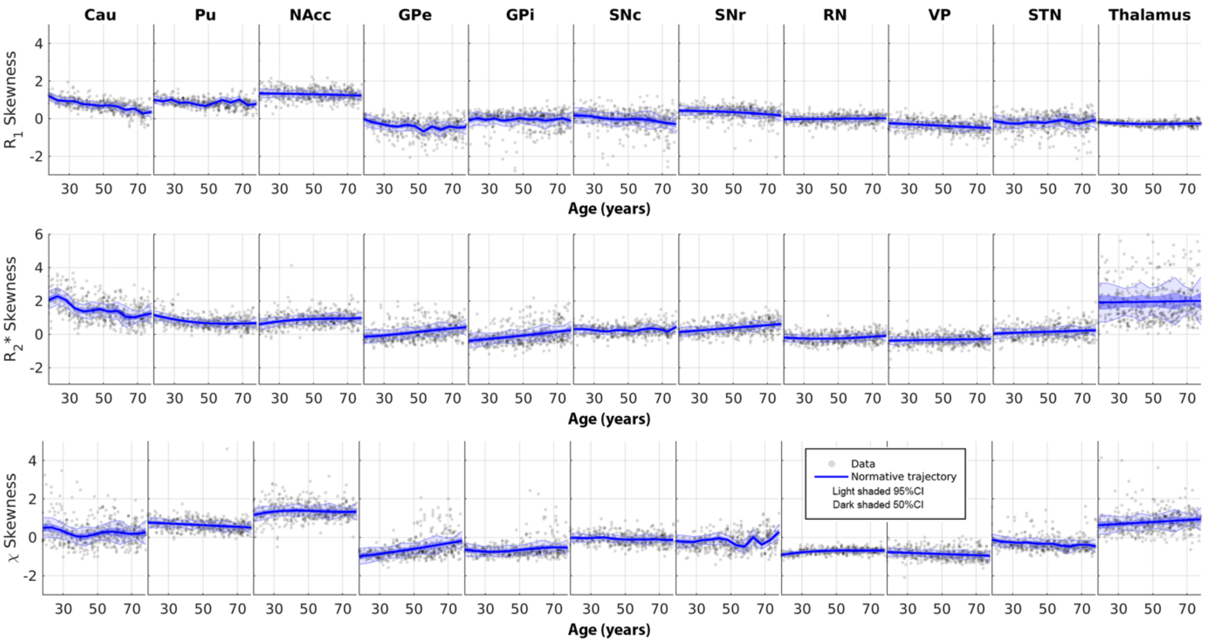

Figure S8: Normative trajectories of the skewness of the distribution of R_1, R_2*, χ as a function of age for 11 different deep grey matter structures present in the MuSus-100 atlas. The data points shown on the scatter plot are corrected for sex and hemispheric effects.

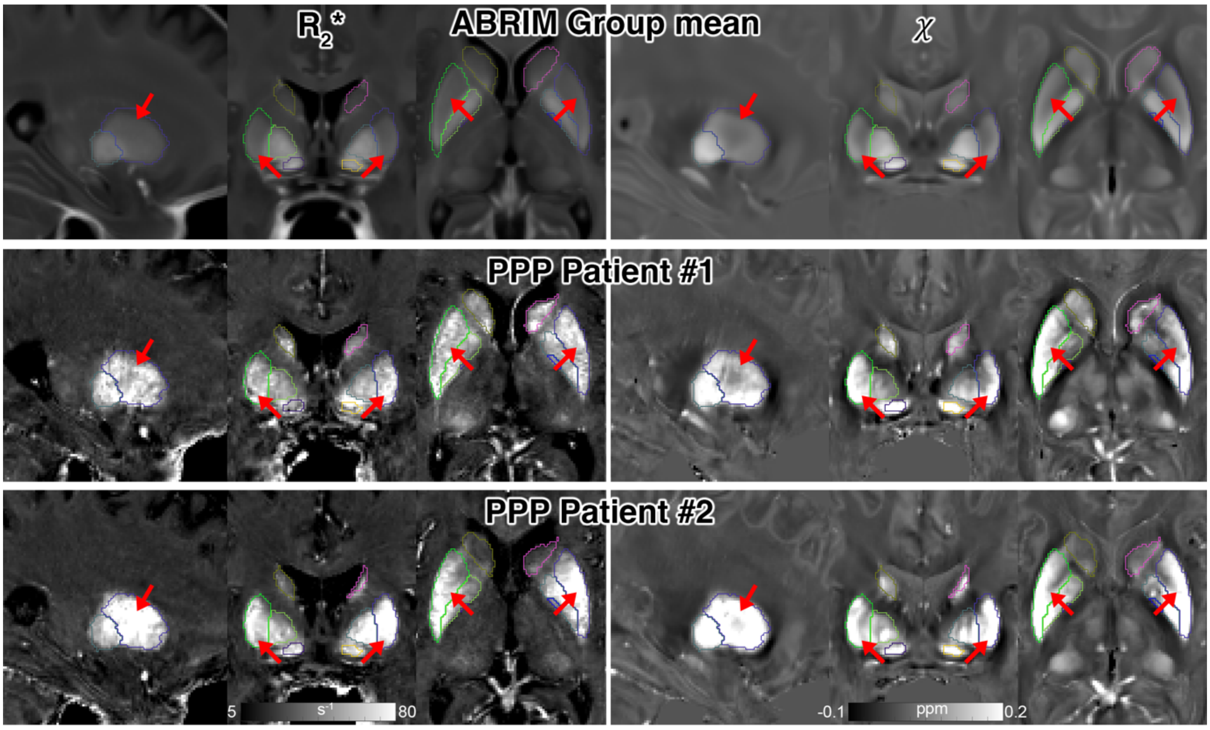

Figure S9: R_2* and χ maps in the subcortical regions of two Parkinson's disease patients with the highest z-scores in the putamen (based on ABRIM normative data). Red arrows indicate the high R_2* and χ values in the putamen in contrast to the ABRIM group averaged maps. Because these offsets are observed on both quantitative maps, an artefactual origin can be readily discarded.

Reference

Alkemade, A., Mulder, M.J., Groot, J.M., Isaacs, B.R., Berendonk, N. van, Lute, N., Isherwood, S.J., Bazin, P.-L., Forstmann, B.U., 2020. The Amsterdam Ultra-high field adult lifespan database (AHEAD): A freely available multimodal 7 Tesla submillimeter magnetic resonance imaging database. Neuroimage 221, 117200. https://doi.org/10.1016/j.neuroimage.2020.117200

Chen, X., Lu, P.-J., Ocampo-Pineda, M., Weigel, M., Chan, K.-S., Cagol, A., Zwiers, M., Jansen, M.G., Norris, D.G., Schädelin, S., Barakovic, M., Kuhle, J., Kappos, L., Melie-Garcia, L., Granziera, C., Marques, J.P., 2024. Normative trajectories of R1, R2*, and Susceptibility values of the healthy human brain cortex, in: Proceedings 32. Annual Meeting International Society for Magnetic Resonance in Medicine. Singapore, p. 0576.

Jansen, M.G., Zwiers, M.P., Marques, J.P., Chan, K.-S., Amelink, J.S., Altgassen, M., Oosterman, J.M., Norris, D.G., 2024. The Advanced BRain Imaging on ageing and Memory (ABRIM) data collection: Study design, data processing, and rationale. PLOS ONE 19, e0306006. https://doi.org/10.1371/journal.pone.0306006

Jenkinson, M., Beckmann, C.F., Behrens, T.E.J., Woolrich, M.W., Smith, S.M., 2012. FSL. Neuroimage 62, 782–790. https://doi.org/10.1016/j.neuroimage.2011.09.015

Marques, J.P., Gruetter, R., 2013. New Developments and Applications of the MP2RAGE Sequence - Focusing the Contrast and High Spatial Resolution R1 Mapping. PLoS ONE 8, e69294. https://doi.org/10.1371/journal.pone.0069294

Marques, J.P., Kober, T., Krueger, G., Zwaag, W. van der, Moortele, P.F.V. de, Gruetter, R., 2010. MP2RAGE, a self bias-field corrected sequence for improved segmentation and T1-mapping at high field. Neuroimage 49, 1271–1281. https://doi.org/10.1016/j.neuroimage.2009.10.002
